# Effects of Dietary Defatted Meat Species on Metabolomic Profiles of Murine Liver, Gastrocnemius Muscle, and Cecal Content

**DOI:** 10.3390/metabo10120503

**Published:** 2020-12-09

**Authors:** Rise Nakata, Mikako Sato, Shozo Tomonaga

**Affiliations:** 1 Graduate School of Agriculture, Kyoto University, Kyoto 606-8502, Japan; riichin37@yahoo.co.jp; 2 Research and Development Center, NH Foods Ltd., Ibaraki 300-2646, Japan; m.satou@nipponham.co.jp

**Keywords:** meat species, metabolomics, liver, muscle, cecal content, carnosine

## Abstract

In both humans and animals, meat not only constitutes one of the sources of protein, but also includes various water-soluble bioactive substances such as imidazole peptides (carnosine and anserine) and taurine. Previous studies demonstrated that dietary meat species could differently affect physiological functions; however, the mechanisms of this remain unclear. To explore the physiological effects of dietary defatted meat species, especially on metabolism, we investigated their influence on the metabolomic profiles of the liver, gastrocnemius muscle, and cecal content in mice. Casein (control) or a defatted meat species (beef leg, pork leg, chicken leg, or chicken breast) was supplied as the major protein source in the diet for four weeks, and metabolism-related molecules were measured by gas chromatography–mass spectrometry. We found that various metabolite levels in tissues and cecal content differed according to the types of dietary protein consumed. Specifically, differences in carnosine, 1,5-anhydro-glucitol, inositol, butyric acid, and propionic acid were clearly observed. Among them, the highest carnosine intake by dietary pork leg was clearly related to the highest carnosine level in the liver. In addition, taurine intake was suggested to be linked to some metabolic pathways including taurine and hypotaurine metabolism in cecal content. These results provide additional knowledge of the effects of different dietary meat species on human and animal health.

## 1. Introduction

The intricate relationship between dietary habits and health is well known, including the role of diet in obesity and metabolic disorders in both humans and animals. Dietary proteins have various effects depending on their origin and quantity [1,2]. Especially in humans, meat is one of the major sources of dietary protein; as such, much attention has been paid to the relationship between meat consumption and health [3,4].

In addition to being a source of protein, meat also includes water-soluble bioactive substances such as imidazole peptides (carnosine and anserine) [5,6] and taurine [7]. Carnosine, a dipeptide composed of β-alanine and _L_-histidine, has antioxidant properties [8], buffering capacity [9], anti-inflammatory activity [10], anti-depressant-like effects [11], and ameliorative effects on the manifestations of metabolic syndrome [12]. Anserine, a dipeptide composed of β-alanine and 1-methyl-L-histidine, also has antioxidant properties [8] and buffering capacity [9]. Taurine is an antioxidant [13] and has anti-depressant properties [14].

The dietary effects of meat species have previously been investigated. Nagasawa et al. [15] compared the dietary effects of soy protein and defatted beef, pork, and chicken in mice, and found that monoamine metabolism in the brain was influenced by the type of dietary meat. They suggested that these effects were independent of the amino acid composition of dietary protein sources. Another study found that dietary chicken or mutton protein induced stronger thermogenesis than did beef, pork, or horse in rats [16]; this was also independent of the amino acid composition of the dietary protein sources. However, neither of these studies clarified the mechanisms involved in eliciting these observed differences. Known and/or unknown bioactive substances may be involved in these actions. Because dietary components can interact with various metabolic pathways in the body, we speculated that using a metabolomics-based approach would be effective in elucidating the various effects of dietary meat sources on bodily functions.

Therefore, we performed this study to investigate dietary effects of defatted meat species on the metabolomic profiles of the liver, gastrocnemius muscle, and cecal content of mice. For analytical samples, liver, gastrocnemius muscle, and cecal content were selected because they have various important roles in the metabolism and/or health of animals.

## 2. Results

### 2.1. Growth Parameters and Tissue Weights

Casein (control) or defatted meat species (beef leg, pork leg, chicken leg, or chicken breast) were fed to mice as their major dietary protein sources for four weeks. We found that the body weights, total food intake, and weights of the liver, brain, lung, gastrocnemius muscle, inguinal fat, epididymal fat, perirenal fat, and brown fat did not differ according to the ingested dietary protein type (Table S1).

### 2.2. Targeted and Non-Targeted Metabolomic Analysis

#### 2.2.1. Quantitative Analysis of Free Amino Acids in the Liver and Gastrocnemius Muscle

Of the 25 amino acids quantified in the liver, the levels of six (alanine, 2-aminoadipic acid, 2-aminobutyric acid, glutamic acid, glycine, and proline) were influenced by the type of dietary protein (Table S2). These six amino acid levels clearly differed between mice fed casein and those fed meat from any source; however, they did not differ according to the type of ingested meat protein.

Of the 24 amino acids quantified in the gastrocnemius muscle, the levels of eight (glycine, isoleucine, leucine, methionine, phenylalanine, proline, tyrosine, and valine) were influenced by the source of dietary protein (Table S3). The levels of all eight amino acids differed according to protein source (casein versus some of meat), although no differences were observed among the types of meat.

#### 2.2.2. Quantitative Analysis of Short-Chained Fatty Acids in Cecal Content

The levels of three short-chained fatty acids were quantified in the cecal content (Table 1). Acetic acid levels were not influenced by the source of dietary protein; however, with propionic acid and butyric acid levels, some differences were confirmed in mice fed casein versus those fed meat. Moreover, propionic acid and butyric acid levels partly differed according to the type of meat ingested.

#### 2.2.3. Non-Targeted and Semi-Quantified Analyses of Metabolites in the Liver, Gastrocnemius Muscle, and Cecal Content

Among the water-soluble bioactive substances in meat, we could not analyze anserine in all samples because the metabolome database we used did not contain analytical conditions for anserine.

Of the 148 metabolites semi-quantified in the liver, the levels of 25 were influenced by the dietary protein source (Table S5). The levels of 19 metabolites differed in mice fed casein versus those fed meat (Table S5); moreover, the levels of 11 metabolites differed according to the type of ingested meat (1,5-anhydro-glucitol, 2-hydroxyisovaleric acid, β-alanine, carnosine, dimethylglycine, glyoxylic acid, inositol, hydroxyproline, sorbitol, tartaric acid, and xylitol) (Table 2). Liver taurine levels were not altered by the diets (Table 2).

Of the 133 metabolites semi-quantified in the gastrocnemius muscle, the levels of seven were influenced by the dietary protein source (Table S6). The levels of six metabolites differed in mice fed casein versus those fed meat (Table S6); moreover, the levels of two metabolites (1,5-anhydro-glucitol and inositol) differed according to the type of meat ingested (Table 3). Levels of β-alanine, carnosine, and taurine in the gastrocnemius muscle were not influenced by the type of diet (Table 3).

Of the 141 metabolites semi-quantified in cecal content, 47 were influenced by the dietary protein source (Table S7). The levels of 35 metabolites differed in mice fed casein versus those fed meat (Table S7), whereas the levels of 18 metabolites were influenced by the type of meat ingested (2-aminobutyric acid, 3-hydroxypropionic acid, 3-methyl-2-oxovaleric acid, 3-sulfinoalanine, β-alanine, carnosine, galactose, glutaric acid, glycerol 3-phosphate, glycine, homocysteine, mannose 6-phosphate, *N*-acetylaspartic acid, *N*-acetylglutamine, nicotinamide, pantothenic acid, phenylacetic acid, and ribose) (Table 4). Taurine was undetectable in cecal content (Table 4).

#### 2.2.4. Integrated Analyses of Quantified and Semi-Quantified Metabolites

Next, data from the aforementioned analyses (Table 1, Table 2, Table 3 and Table 4, Table S2, Table S3, Tables S5–S7) were integrated and reanalyzed. The results were as follows:

Of 173 metabolites tested in the liver, 31 were influenced by the source of dietary protein. The levels of 25 metabolites differed in mice fed casein versus those fed meat; moreover, the levels of 11 metabolites differed according to the type of ingested meat (1,5-anhydro-glucitol, 2-hydroxyisovaleric acid, β-alanine, carnosine, dimethylglycine, glyoxylic acid, inositol, hydroxyproline, sorbitol, tartaric acid, and xylitol).

Of 157 metabolites tested in the gastrocnemius muscle, the levels of 15 were influenced by the dietary protein source. The levels of 14 metabolites differed in mice fed casein versus those fed meat, while the levels of two differed according to the type of meat (1,5-anhydro-glucitol and inositol).

Of the total 144 metabolites tested in cecal content, 49 were influenced by the dietary protein source. The levels of 37 metabolites difference in mice fed casein versus those fed meat; moreover, the levels of 20 metabolites differed according to the type of meat ingested (2-aminobutyric acid, 3-hydroxypropionic acid, 3-methyl-2-oxovaleric acid, 3-sulfinoalanine, β-alanine, butyric acid, carnosine, galactose, glutaric acid, glycerol 3-phosphate, glycine, homocysteine, mannose 6-phosphate, *N*-acetylaspartic acid, *N*-acetylglutamine, nicotinamide, pantothenic acid, phenylacetic acid, propionic acid, and ribose).

**Figure 1:**
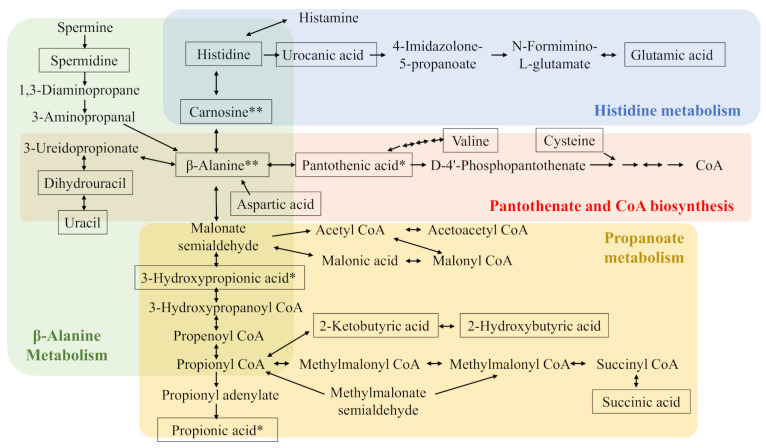
Carnosine-related metabolism. Boxed metabolites are quantified or semi-quantified metabolites in the liver, gastrocnemius muscle, or cecal content. * indicates metabolites affected by meat species in cecal content. ** indicates metabolites affected by meat species in both cecal content and liver. CoA, coenzyme A.

Analyses of the pathways associated with the significantly affected metabolites revealed that 12, 6, and 14 pathways in the liver, gastrocnemius muscle, and cecal content, respectively, were potentially affected by diet (Table 5). Metabolic pathway number (Metab. No., Table S4) was added to each pathway in Table 5. Metab. Nos. 6, 7, 15, and 20 are related to carnosine metabolism while the Nos. 5 and 22 are related to taurine metabolism. These were added to their corresponding metabolites shown in Table 1, Table 2, Table 3 and Table 4. As a result, the effects of dietary meat species on carnosine-related metabolism, not taurine-related metabolism, were clearly confirmed. They were summarized in Figure 1.

To explore differences among dietary meat species further, partial least squares discriminant analysis (PLS-DA) was tried for four different meat groups using metabolite levels in liver, gastrocnemius muscle, or cecal content. However, no good discriminant model could be made (data not shown).

#### 2.2.5. Carnosine, Anserine, and Taurine Levels in Experimental Protein Sources

Among the water-soluble bioactive substances in meat, carnosine levels were clearly affected by the type of dietary meat ingested (Table 1, Table 2, Table 3, Table 4 and Table 5, Figure 1). Therefore, we quantified water-soluble bioactive substances in meat including carnosine in all protein sources (Table 6). Carnosine, anserine, and taurine were not detected in casein, but were detected at markedly different levels in meat species.

Next, intake of carnosine, anserine, and taurine (Table 7) were calculated and analyzed by their levels in experimental protein sources (Table 6), diet composition (Table S8), and food intake (Table S1). The highest intake of carnosine was detected in pork leg and the lowest in chicken leg. Interestingly, the same patterns were observed in the liver and cecal content (Table 2 and Table 4) but not in muscle (Table 3). Then, to explore metabolic effects by intake of carnosine, anserine, or taurine, PLS regression (PLS-R) was tried using metabolite levels in liver, gastrocnemius muscle, or cecal content. Consequently, only one good regression model could be made, which can predict taurine intake by metabolite levels of cecal content (R^2^Y = 0.996, Q^2^ = 0.82). Analysis of pathways associated with the significant metabolites revealed that 13 metabolic pathways were potentially affected by taurine intake (Table 8).

## 3. Discussion

There were no obvious detrimental effects of meat-based diets on growth parameters (food intake, body weight, and tissue weights) when compared to a casein-based diet (the control). On the other hand, the levels of numerous metabolites were influenced by the source of the dietary protein. Moreover, pathway analyses suggested that certain metabolic pathways were significantly affected by the type of protein in the diet. Significant differences in the levels of numerous metabolites were observed in mice fed casein versus meat; such differences may be a result of the amino acid composition of the proteins (Table S9) and/or the existence of meat-specific bioactive substances. While we mainly focused on the metabolic effects of various dietary meat species, the intra-meat differences did not appear to be as marked as those between casein and meat given that the compositions of amino acids among the various meat species were almost identical (Table S9). Therefore, we investigated meat-derived bioactive substances that were associated with obviously different metabolite levels (Table 6).

Carnosine-related metabolism was clearly affected by the dietary meat species. Higher dietary carnosine intake resulted in both higher carnosine and β-alanine levels in the liver and cecal content. These results are consistent with our previous study that showed that dietary carnosine increased both carnosine and β-alanine levels in the liver and cecal content of mice in a dose-dependent manner [17]. The increased carnosine levels may have a positive effect on animal health as stated in the Introduction. On the other hand, a limitation of the present study is that we could not fully evaluate the effects of dietary anserine levels on anserine-related metabolism because present analytical methods could not confirm the presence of anserine and its metabolite 1-methylhistidine in tissues and cecal content. Because anserine also has various actions (some in common with carnosine) [8,9], its role should be evaluated in future studies. On the other hand, 2-oxo-imidazole dipeptides could be produced from imidazole peptides in mouse tissue and have strong antioxidant activity [18]. Therefore, to evaluate dietary effects obtained in the present study more precisely, not only carnosine and anserine, but also 2-oxo-carnosine and 2-oxo-anserine should be investigated in the future in this mouse model.

We observed no influence of dietary taurine on taurine levels in tissues and cecal content, indicating that dietary taurine levels may not be sufficient to elicit an increase in free taurine expression. On the other hand, effects of taurine intake on some metabolic pathways including taurine and hypotaurine metabolism were speculated by the pathway analysis in cecal content. To evaluate metabolic effects of dietary taurine further, focusing on not only taurine metabolic pathway but also other metabolic pathways suggested in the present study may be effective.

In terms of tissues, 1,5-anhydro-glucitol and inositol were affected by dietary meat species in both liver and muscle. Because dietary 1,5-anhydro-glucitol can modulate glucose homeostasis [19] while dietary inositol can regulate lipid metabolism [20], our current results may provide useful information with which to understand the effects of dietary meat species on metabolic syndrome, given that it is closely linked to glucose and lipid metabolism.

Butyric and propionic acid levels in cecal content were affected by the type of dietary meat species. These short-chain fatty acids have important roles in animal health, including metabolic syndrome [21]; therefore, our results ought to be informative for understanding the different effects of dietary meat species. Propionic acid can be derived from β-alanine (Figure 1), and its highest levels were observed in mice fed chicken breast, which contains the highest levels of imidazole dipeptides (the combination of carnosine and anserine). Therefore, propionic acid levels may be, in part, derived from β-alanine resulting from the degradation of dietary carnosine and/or anserine. To better investigate this, additional studies such as metabolic flux analysis should be performed.

In conclusion, we identified some metabolites whose levels in tissues or cecal content differed according to the type of dietary meat species. These included some bioactive substances such as carnosine, 1,5-anhydro-glucitol, inositol, butyric acid, and propionic acid. In addition, some metabolic pathways linked to taurine intake could be suggested. Focusing on these results will be useful to clarify the different effects of dietary meat species on metabolism and/or health in humans and animals.

## 4. Materials and Methods

### 4.1. Animals and Diets

Thirty male C57BL/6J mice (three weeks old, SLC Japan) were used in this experiment. The animals were housed individually in plastic cages in a room maintained at 24 ± 1 °C with a 14 h–10 h light–dark cycle (the lights were on from 5:00 to 19:00). All animals were allowed free access to water and were fed a normal diet (AIN-93G) for one week before the experiment. Institutional ethical approval was obtained (code number 29–87).

The ingredients of the AIN-93G diet used in this study are shown in Table S8; this diet uses casein (CLEA Japan Inc., Tokyo, Japan) as the protein source. Casein was replaced with powdered and defatted meat including from beef leg, pork leg, chicken leg, or chicken breast for use as experimental diets. Animal meats (beef leg, pork leg, chicken leg, and chicken breast) were prepared at NH Foods Ltd. (Osaka, Japan). After removing the fat, tendons, and peels, each type of meat was packed in vacuo and heated for 60 min at 75 °C in a convection oven. After confirming that the temperature at the center of the pack was over 72 °C, meats were cooled in iced water. The powder of each meat was produced according to the method of Wakamatsu et al. [22]; in brief, all the meats were minced and freeze-dried, and fat was removed using n-hexane. Table S9 shows the amino acid components of the experimental protein sources. Other food materials were purchased from CLEA Japan Inc. (Tokyo, Japan). We prepared each diet by mixing these food materials.

After acclimation, mice were divided into five groups based on body weight and were fed their assigned diets for four weeks. Daily food intake and weekly body weight were recorded during the experimental period. At the end of the experiment, blood was drawn under anesthesia and plasma was isolated by centrifugation at 10,000× *g* and 4 °C for 10 min. The brain, liver, gastrocnemius muscle, inguinal fat, epididymal fat, perirenal fat, and brown fat were removed and their wet weights were measured. Additionally, cecal content was removed and all samples were immediately frozen in liquid nitrogen and stored at −80 °C. The liver and gastrocnemius muscle were crushed with liquid nitrogen and powdered before the analysis.

### 4.2. Gas Chromatography/Mass Spectrometry (GC–MS/MS)-Based Non-Targeted Metabolomic Analysis

Cecal content were diluted five-fold in distilled water and centrifuged at 20,000× *g* and 4 °C for 5 min. The resulting supernatant (50 μL), along with liver (10 mg) and gastrocnemius muscle (10 mg), underwent metabolomic analysis. The samples were treated according to Goto et al. [23]; briefly, the hydrophilic fraction was extracted and derivatized by methoxyamine hydrochloride (MP Biomedicals, California, USA) and *N*-methyl-*N*-(trimethylsilyl)trifluoroacetamide (Funakoshi, Tokyo, Japan). Moreover, 2-isopropylmalic acid was used as an internal standard. GC–MS/MS analyses were performed using a GCMS-TQ8050 instrument (Shimadzu, Kyoto, Japan). According to Smart Metabolites Database (Shimadzu, Kyoto, Japan), a DB-5 column (length 30 m, inner diameter 0.25 mm, and film thickness 1.00 μm) (Agilent Technologies, Santa Clara, CA, USA) was used for the liver and gastrocnemius muscle, while a BPX-5 column (length 30 m, inner diameter 0.25 mm, and film thickness 0.25 μm) (SGE, Melbourne, Australia) was used for cecal content.

Data processing was performed using the Smart Metabolites Database, MS-DIAL version 3.04 [24] and MRMPROBS program version 2.50 [25]. An annotation of metabolites was conducted using thresholds as follows: −10 < retention index < +10, a mass spectrum similarity of >50 in the SCAN data, and the presence of a second ion peak in the Multiple Reaction Monitoring (MRM) data according to the analysis using the Smart Metabolites Database and MS-DIAL. The relative level of a metabolite was calculated using the peak area of each metabolite relative to that of the internal standard (2-isopropylmalic acid) using the MRMPROBS program version 2.50. The value of each metabolite was then corrected so that the average value of all samples was 100.

### 4.3. GC–MS-Based Free Amino Acid Analysis

For amino acid analysis, 300 μL of 0.1 M perchloric acid was added to liver, gastrocnemius muscle, and cecal content (30 mg each) and homogenized and placed on ice in a dark place for 30 min. These were then centrifugated (20,000× *g*, 0 °C) for 15 min to obtain the supernatant. Norvaline as internal standard was added to this supernatant and mixed amino acids standard. After further treatment and derivatization using the EZfaast kit (Phenomenex, CA, USA), these samples were analyzed in the SIM mode using the GCMS-QP2010 Ultra (Shimadzu, Kyoto, Japan) with a Zebron ZB-AAA capillary GC column (length 10 m, inner diameter 0.25 mm) (Phenomenex, Torrance, CA, USA) according to the Smart Metabolites Database. Amino acid levels were calculated using the peak area of each metabolite relative to that of the internal standard (norvaline).

### 4.4. GC–MS/MS-Based Short-Chain Fatty Acid Analysis

Cecal content (50 mg) was diluted in distilled water (200 μL) and centrifuged at 20,000× *g* and 4 °C for 5 min. For an internal standard, acetic acid-d4 was added to this supernatant (50 μL) and to mixed short-chain fatty acids (acetic acid, propionic acid, and butyric acid). After adding methanol and shaking for 30 s, the solution was centrifuged at 16,000× *g* and 25 °C for 3 min. The supernatant (180 μL) was obtained and derivatized using 4-(4,6-dimethoxy-1,3,5-triazin-2-yl)-4-methylmorpholinium chloride methanol solution and n-octylamine methanol solution for 9 h. These samples were then analyzed in MRM mode using the GCMS-TQ8050 with a BPX-5 column (length 30 m, inner diameter 0.25 mm, and film thickness 0.25 μm). Helium was used as the carrier gas at a pressure of 68.4 kPa, with a total flow rate of 40.4 mL/min and purge flow rate of 5.0 mL. The splitless injection mode was used. The column oven temperature program was to hold at 60 °C for 2 min, then increase at 15 °C per min from 60 to 330 °C. The vaporization room and ion source temperatures were 250 °C and 200 °C, respectively. Each metabolite was quantified using its peak area relative to that of the internal standard (acetic acid-d4).

### 4.5. Amino Acid Composition, Imidazole Dipeptides, and Taurine Quantification in Protein Sources

The amino acid content in hydrochloric-acid-hydrolyzed protein sources was quantified using an amino acid analyzer JLC-500/V (JEOL, Tokyo, Japan). Imidazole dipeptides (carnosine and anserine) and taurine levels in sulfosalicylic-acid-deproteinized protein sources were also quantified using the JLC-500/V analyzer.

### 4.6. Statistical Analyses

Data are expressed as means ± standard error of the mean. Data comparisons were performed using one-way analysis of variance with Tukey’s post-hoc multiple comparison test. The JMP software version 13.0.0 (SAS Institute, Cary, NC, USA) was used to perform these analyses. Metabolites significantly affected were subjected to pathway analysis using MetaboAnalyst 4.0 [26]. “*Mus musculus* (Kyoto Encyclopedia of Genes and Genomes)” was used for the pathway library. In these analyses, *p* < 0.05 was considered statistically significant. For PLS-DA and PLS-R, SIMCA 13.0.3 (Umetrics, Umeå, AB, Sweden) was used. In PLS-R, significant metabolite was determined as its variable importance in projection (VIP) score is larger than one and larger than the cross-validation standard error.

## Figures and Tables

**Table 1:** Effects of dietary protein sources on short-chain fatty acid levels in the cecal content of mice.

	Casein	Beef Leg	Pork Leg	Chicken Leg	Chicken Breast	ANOVA	Metab. No.
Acetic acid	20.42 ± 0.97	17.24 ± 1.85	18.00 ± 1.98	23.29 ± 1.42	21.19 ± 1.38	NS	-
Propionic acid	3.48 ± 0.28 ^a^	1.96 ± 0.30 ^b^	1.97 ± 0.32 ^b^	3.76 ± 0.24 ^a^	3.44 ± 0.58 ^a,b^	<0.05	6
Butyric acid	7.09 ± 0.42 ^b^	8.7 ± 0.93 ^a,b^	8.94 ± 0.37 ^a,b^	7.83 ± 0.36 ^b^	10.73 ± 0.91 ^a^	<0.05	-

Values (μmol/g) are means with their standard errors (*n* = 6). NS, not significant (*p* ≥ 0.05); ANOVA, analysis of variance; Metab. No., metabolic pathway number for carnosine-related metabolism (6, 7, 15, and 20) or taurine-related metabolism (5 and 22) shown in Table S4. Different letters in the same line denote significantly different mean values according to the Tukey test (*p* < 0.05).

**Table 2:** Effects of dietary protein sources on metabolite levels in the liver of mice.

	Casein	Beef Leg	Pork Leg	Chicken Leg	Chicken Breast	ANOVA	Metab. No.
1,5-Anhydro-glucitol	111 ± 4 ^a,b^	97 ± 5 ^b,c^	129 ± 8 ^a^	87 ± 4 ^c^	76 ± 5 ^c^	<0.0001	-
2-Hydroxyisovaleric acid	128 ± 9 ^a^	80 ± 3 ^c^	105 ± 5 ^a,b^	99 ± 2 ^b,c^	88 ± 6 ^b,c^	<0.0001	-
β-Alanine	85 ± 5 ^c^	88 ± 3 ^b,c^	117 ± 4 ^a^	100 ± 5 ^a,b,c^	111 ± 9 ^a,b^	<0.05	6, 7, 15, 20
Carnosine	7 ± 1 ^d^	125 ± 21 ^b^	242 ±30 ^a^	39 ± 5 ^c,d^	86 ± 19 ^b,c^	<0.0001	7, 15
Dimethylglycine	90 ± 10 ^a,b^	115 ± 14 ^a,b^	83 ± 10 ^a,b^	76 ± 4 ^b^	136 ± 24 ^a^	<0.05	-
Glyoxylic acid	91 ± 10 ^a,b^	114 ± 14 ^a,b^	83 ± 9 ^a,b^	76 ± 3 ^b^	136 ± 25 ^a^	<0.05	-
Inositol	82 ± 4 ^b^	96 ± 4 ^b^	101 ± 2 ^a,b^	121 ± 4 ^a^	101 ± 8 ^a,b^	<0.05	-
Hydroxyproline	98 ± 4 ^b^	91 ± 2 ^b^	104 ± 4 ^a,b^	120 ± 7 ^a^	87 ± 5 ^b^	<0.05	-
Sorbitol	86 ± 13 ^b^	86 ± 14 ^b^	92 ± 11 ^a,b^	137 ± 11 ^a^	98 ± 9 ^a,b^	<0.05	-
Tartaric acid	61 ± 8 ^c^	95 ± 14 ^a,b,c^	129 ±21 ^a,b^	143 ± 21 ^a^	72 ± 7 ^b,c^	<0.05	-
Taurine	95 ± 6	94 ± 5	105 ± 3	110 ± 4	97 ± 7	NS	22
Xylitol	95 ± 4 ^a,b^	93 ± 6 ^b^	100 ± 2 ^a,b^	115 ± 4 ^a^	96 ± 7 ^a,b^	<0.05	-

Relative values are means with their standard errors (*n* = 6). NS, not significant (*p* ≥ 0.05); ANOVA, analysis of variance; Metab. No., metabolic pathway number for carnosine-related metabolism (6, 7, 15, and 20) or taurine-related metabolism (5 and 22) shown in Table S4. Different letters in the same line denote significantly different mean values according to the Tukey test (*p* < 0.05).

**Table 3:** Effect of dietary protein sources on metabolite levels in the gastrocnemius muscle of mice.

	Casein	Beef Leg	Pork Leg	Chicken Leg	Chicken Breast	ANOVA	Metab. No.
1,5-Anhydro-glucitol	110 ± 9 ^a,b^	94 ± 2 ^b^	123 ± 5 ^a^	88 ± 5 ^b^	86 ± 7 ^b^	<0.05	-
β-Alanine	100 ± 10	96 ± 10	104 ± 10	98 ± 6	102 ± 10	NS	6, 7, 15, 20
Carnosine	119 ± 21	81 ± 17	86 ± 14	105 ± 22	108 ± 26	NS	7, 15
Inositol	83 ± 9 ^b^	104 ± 4 ^a,b^	97 ± 4 ^b^	123 ± 6 ^a^	93 ± 3 ^b^	<0.05	-
Taurine	99 ± 3	99 ± 1	101 ± 2	102 ± 4	100 ± 1	NS	22

Relative values are means with their standard errors (*n* = 6). NS, not significant (*p* ≥ 0.05); ANOVA, analysis of variance; Metab. No., metabolic pathway number for carnosine-related metabolism (6, 7, 15, and 20) or taurine-related metabolism (5 and 22) shown in Table S4. Different letters in the same line denote significantly different mean values according to the Tukey test (*p* < 0.05).

**Table 4:** Effect of dietary protein sources on metabolite levels in the cecal content of mice.

	Casein	Beef Leg	Pork Leg	Chicken Leg	Chicken Breast	ANOVA	Metab. No.
2-Aminobutyric acid	114 ± 12 ^a,b^	72 ± 9 ^b^	65 ± 13 ^b^	147 ± 26 ^a^	103 ± 23 ^a,b^	<0.05	5
3-Hydroxypropionic acid	83 ± 6 ^b^	127 ± 8 ^a^	90 ± 3 ^b^	104 ± 9 ^a,b^	97 ± 9 ^a,b^	<0.05	6, 7
3-Methyl-2-oxovaleric acid	80 ± 8 ^b^	82 ± 3 ^b^	99 ± 14 ^ab^	138 ± 12 ^a^	101 ± 21 ^a,b^	<0.05	-
3-Sulfinoalanine	55 ± 8 ^b^	88 ± 14 ^b^	103 ± 15 ^a,b^	167 ± 27 ^a^	87 ± 14 ^b^	<0.05	5, 22
β-Alanine	20 ± 3 ^b^	268 ± 30 ^a^	86 ± 14 ^b^	42 ± 4 ^b^	83 ± 18 ^b^	<0.0001	6, 7, 15, 20
Carnosine	0 ± 0 ^b^	289 ± 53 ^a^	180 ± 33 ^a^	4 ± 1 ^b^	27 ± 11 ^b^	<0.0001	7, 15
Galactose	148 ± 39 ^a^	48 ± 7 ^b^	77 ± 10 ^a,b^	153 ± 29 ^a^	74 ± 8 ^a,b^	<0.05	-
Glutaric acid	85 ± 16 ^b^	154 ± 20 ^a^	104 ± 10 ^a,b^	83 ± 5 ^b^	74 ± 5 ^b^	<0.05	-
Glycerol 3-phosphate	74 ± 13 ^a,b^	116 ± 17 ^a,b^	129 ± 14 ^a^	62 ± 14 ^b^	118 ± 18 ^a,b^	<0.05	-
Glycine	106 ± 13 ^a,b^	97 ± 16 ^a,b^	72 ± 13 ^b^	137 ± 13 ^a^	88 ± 9 ^ab^	<0.05	-
Homocysteine	134 ± 16 ^a^	87 ± 12 ^a,b^	100 ± 9 ^a,b^	61 ± 4 ^b^	117 ± 15 ^a^	<0.05	5
Mannose 6-phosphate	64 ± 14 ^b^	94 ± 14 ^a,b^	124 ± 21 ^a,b^	68 ± 19 ^b^	151 ± 26 ^a^	<0.05	-
*N*-Acetylaspartic acid	33 ± 8 ^c^	56 ± 11 ^b,c^	142 ± 28 ^a,b^	163 ± 22 ^a^	107 ± 36 ^a,b,c^	<0.05	-
*N*-Acetylglutamine	32 ± 7 ^c^	30 ± 4 ^c^	150 ± 32 ^a,b^	200 ± 13 ^a^	89 ± 40 ^b,c^	<0.0001	-
Nicotinamide	16 ± 1 ^d^	106 ± 6 ^b^	226 ± 13 ^a^	86 ± 5 ^bc^	65 ± 12 ^c^	<0.0001	-
Pantothenic acid	120 ± 18 ^a,b^	77 ± 8 ^b^	70 ± 7 ^b^	137 ± 18 ^a^	95 ± 13 ^a,b^	<0.05	20
Phenylacetic acid	42 ± 5 ^b^	97 ± 10 ^b^	100 ± 18 ^b^	174 ± 17 ^a^	86 ± 16 ^b^	<0.0001	-
Ribose	102 ± 10 ^a,b^	74 ± 8 ^b^	120 ± 5 ^a^	109 ± 8 ^a,b^	95 ± 13 ^a,b^	<0.05	-
Taurine	ND	ND	ND	ND	ND	-	22

Relative values are means with their standard errors (*n* = 6). ND, not detected; ANOVA, analysis of variance; Metab. No., metabolic pathway number for carnosine-related metabolism (6, 7, 15, and 20) or taurine-related metabolism (5 and 22) shown in Table S4. Different letters in the same line denote significantly different mean values according to the Tukey test (*p* < 0.05).

**Table 5:** Potential metabolic pathways affected by dietary protein sources.

Metab. No.	Pathway Name	Liver	Gastrocnemius Muscle	Cecal Content
1	Aminoacyl-tRNA biosynthesis	*	**	**
2	Glycine, serine, and threonine metabolism	*	*	*
3	Valine, leucine, and isoleucine biosynthesis		**	**
4	Valine, leucine, and isoleucine degradation		*	*
5	Cysteine and methionine metabolism	*		*
6	Propanoate metabolism	*		*
7	β-Alanine metabolism	*		*
8	Fructose and mannose metabolism	*		*
9	Glycerolipid metabolism	*		*
10	Alanine, aspartate, and glutamate metabolism	*		
11	Arginine and proline metabolism	*		
12	Butanoate metabolism	*		
13	Glutamate metabolism	*		
14	Glyoxylate and dicarboxylate metabolism	*		
15	Histidine metabolism	*		
16	Phenylalanine, tyrosine, and tryptophan biosynthesis		*	
17	Phenylalanine metabolism		*	
18	Amino sugar and nucleotide sugar metabolism			*
19	Galactose metabolism			*
20	Pantothenate and CoA biosynthesis			*
21	Pentose phosphate pathway			*
22	Taurine and hypotaurine metabolism			*

* *p* < 0.05; ** *p* < 0.0001. Metab. No., metabolic pathway number shown in Table S4.

**Table 6:** Carnosine, anserine, and taurine levels in experimental protein sources.

	Casein	Beef Leg	Pork Leg	Chicken Leg	Chicken Breast
Carnosine	ND	11,245	25,742	4763	9021
Anserine	ND	2846	1040	10,615	26,601
Taurine	ND	1246	2034	6421	1123

The unit is μg/g. ND, not detected.

**Table 7:** Intake of carnosine, anserine, and taurine in mice fed various meat protein species.

	Beef Leg	Pork Leg	Chicken Leg	Chicken Breast	ANOVA
Carnosine	451 ± 12 ^b^	963 ± 20 ^a^	179 ± 3 ^d^	342 ± 7 ^c^	<0.0001
Anserine	114 ± 3 ^c^	39 ± 1 ^d^	400 ± 7 ^b^	1009 ± 21 ^a^	<0.0001
Taurine	50 ± 1 ^c^	76 ± 2 ^b^	242 ± 4 ^a^	43 ± 1 ^c^	<0.0001

Values (mg) are means with their standard errors (*n* = 6). ANOVA, analysis of variance. Different letters in the same line denote significantly different mean values according to the Tukey test (*p* < 0.05).

**Table 8:** Potential metabolic pathways linked to taurine intake in cecal content.

Metab. No.	Pathway Name	Cecal Content
1	Aminoacyl-tRNA biosynthesis	**
2	Glycine, serine and threonine metabolism	*
3	Valine, leucine and isoleucine biosynthesis	**
5	Cysteine and methionine metabolism	*
6	Propanoate metabolism	*
8	Fructose and mannose metabolism	*
10	Alanine, aspartate and glutamate metabolism	*
14	Glyoxylate and dicarboxylate metabolism	*
16	Phenylalanine, tyrosine and tryptophan biosynthesis	*
17	Phenylalanine metabolism	*
18	Amino sugar and nucleotide sugar metabolism	*
20	Pantothenate and CoA biosynthesis	*
22	Taurine and hypotaurine metabolism	*

* *p* < 0.05; ** *p* < 0.0001. Metab. No., metabolic pathway number shown in Table S4.

## Supplementary Materials

The following are available online at https://www.mdpi.com/2218-1989/10/12/503/s1, Table S1: Growth parameters, Table S2: Liver amino acids, Table S3: Muscle amino acids, Table S4: Number and name of metabolic pathway, Table S5: Liver metabolites, Table S6: Muscle metabolites, Table S7: Cecal content metabolites, Table S8: Diet composition, and Table S9: Amino acid composition.
