# Supplementary material for: Effects of Dietary Defatted Meat Species on Metabolomic Profiles of Murine Liver, Gastrocnemius Muscle, and Cecal Content
Source: Metabolites. 2020 Dec 9;10(12):503. doi: 10.3390/metabo10120503 (PMC7763243; doi:10.3390/metabo10120503)
Supplement: Supplementary file 1 [file metabolites-10-00503-s001.zip › Supplementary Tables/Table S1 Growth parameters.docx]

Table S1 Growth parameters and tissue weights

|  | Casein | Beef  Leg | Pork  Leg | Chicken  Leg | Chicken  Breast | ANOVA |
| --- | --- | --- | --- | --- | --- | --- |
| Food intake (g) | 198± 4 | 197± 5 | 189± 4 | 188± 3 | 192± 4 | NS |
| Initial  Body weight (g) | 16± 1 | 16± 0 | 16± 0 | 16± 0 | 16± 0 | NS |
| Final  Body weight (g) | 28± 1 | 27± 1 | 27± 1 | 29± 1 | 27± 0 | NS |
| Liver (mg) | 1,288±52 | 1,289±40 | 1,317±57 | 1,314±53 | 1,296±46 | NS |
| Gastrocnemius muscle (mg) | 126±13 | 141± 8 | 136± 7 | 138± 5 | 143± 8 | NS |
| inguinal fat (mg) | 143±30 | 111±16 | 101±14 | 133±27 | 119±15 | NS |
| epididymal  fat (mg) | 529±67 | 546±35 | 492±60 | 624±74 | 523±35 | NS |
| perirenal fat (mg) | 204±45 | 152±12 | 153±28 | 203±32 | 146±19 | NS |
| brown fat (mg) | 210±32 | 244±37 | 219±40 | 238±14 | 188±13 | NS |

Values are means with their standard errors (n = 6). NS: not significant (P ≥ 0.05); ANOVA: analysis of variance.
