# Supplementary material for: Effects of Dietary Defatted Meat Species on Metabolomic Profiles of Murine Liver, Gastrocnemius Muscle, and Cecal Content
Source: Metabolites. 2020 Dec 9;10(12):503. doi: 10.3390/metabo10120503 (PMC7763243; doi:10.3390/metabo10120503)
Supplement: Supplementary file 1 [file metabolites-10-00503-s001.zip › Supplementary Tables/Table S2 Liver amino acids.docx]

Table S2 Effects of dietary protein sources on free amino acid levels in the liver

|  | Casein | Beef  Leg | Pork  Leg | Chicken  Leg | Chicken  Breast | ANOVA |
| --- | --- | --- | --- | --- | --- | --- |
| 2-Aminoadipic acid | 0.17±0.03^a^ | 0.06±0.01^b^ | 0.07±0.01^b^ | 0.06±0.01^b^ | 0.05±0.02^b^ | <.0001 |
| Alanine | 6.30±0.32^a^ | 4.51±0.22^b^ | 5.09±0.29^b^ | 4.74±0.34^b^ | 3.94±0.25^b^ | <.0001 |
| Glutamic acid | 1.44±0.12^a^ | 0.91±0.06^b^ | 0.92±0.07^b^ | 0.85±0.08^b^ | 0.83±0.03^b^ | <.0001 |
| Proline | 0.42±0.03^a^ | 0.27±0.01^b^ | 0.28±0.01^b^ | 0.28±0.02^b^ | 0.27±0.01^b^ | <.0001 |
| 2-Aminobutyric acid | 0.02±0.00^a^ | 0.01±0.00^b^ | 0.01±0.00^b^ | 0.01±0.00^b^ | 0.01±0.00^b^ | <0.05 |
| Glycine | 1.64±0.09^b^ | 1.98±0.04^a^ | 2.02±0.08^a^ | 2.18±0.06^a^ | 2.07±0.08^a^ | <0.05 |
| 4-Aminobutyric acid | 0.03±0.00 | 0.02±0.00 | 0.02±0.00 | 0.02±0.00 | 0.03±0.00 | NS |
| Asparagine | 0.21±0.02 | 0.21±0.01 | 0.20±0.02 | 0.20±0.02 | 0.22±0.02 | NS |
| Aspartic acid | 0.43±0.05 | 0.35±0.02 | 0.37±0.08 | 0.34±0.05 | 0.35±0.02 | NS |
| Cystathionine | 0.18±0.02 | 0.15±0.01 | 0.17±0.01 | 0.17±0.01 | 0.14±0.00 | NS |
| Cystine | 0.04±0.01 | 0.06±0.01 | 0.06±0.02 | 0.04±0.01 | 0.05±0.01 | NS |
| Glutamine | 4.68±0.49 | 5.32±0.47 | 5.02±0.65 | 5.43±0.63 | 5.57±0.66 | NS |
| Histidine | 0.85±0.02 | 0.77±0.05 | 0.81±0.07 | 0.79±0.06 | 0.75±0.05 | NS |
| Isoleucine | 0.19±0.02 | 0.17±0.01 | 0.16±0.01 | 0.17±0.02 | 0.18±0.02 | NS |
| Leucine | 0.45±0.06 | 0.40±0.02 | 0.36±0.02 | 0.36±0.04 | 0.39±0.04 | NS |
| Lysine | 1.44±0.14 | 1.12±0.12 | 1.26±0.14 | 1.38±0.16 | 1.20±0.12 | NS |
| Methionine | 0.16±0.01 | 0.16±0.00 | 0.16±0.01 | 0.15±0.01 | 0.15±0.01 | NS |
| Ornithine | 0.69±0.06 | 0.60±0.06 | 0.61±0.06 | 0.73±0.05 | 0.56±0.05 | NS |
| Phenylalanine | 0.23±0.01 | 0.23±0.01 | 0.22±0.01 | 0.22±0.01 | 0.23±0.01 | NS |
| Sarcosine | 0.18±0.01 | 0.16±0.00 | 0.16±0.00 | 0.17±0.00 | 0.16±0.00 | NS |
| Serine | 0.40±0.02 | 0.40±0.03 | 0.35±0.03 | 0.34±0.03 | 0.41±0.04 | NS |
| Threonine | 0.41±0.02 | 0.40±0.03 | 0.37±0.02 | 0.38±0.03 | 0.39±0.04 | NS |
| Tryptophan | 0.20±0.00 | 0.20±0.00 | 0.20±0.00 | 0.20±0.00 | 0.20±0.00 | NS |
| Tyrosine | 0.38±0.02 | 0.32±0.01 | 0.32±0.01 | 0.33±0.02 | 0.33±0.01 | NS |
| Valine | 0.39±0.05 | 0.31±0.02 | 0.29±0.02 | 0.3±0.04 | 0.34±0.04 | NS |

Values (µmol/g) are means with their standard errors (n = 6). NS: not significant (P ≥ 0.05); ANOVA: analysis of variance. Different letters in the same line denote significantly different mean values according to the Tukey test (P < 0.05).
