# Supplementary material for: Effects of Dietary Defatted Meat Species on Metabolomic Profiles of Murine Liver, Gastrocnemius Muscle, and Cecal Content
Source: Metabolites. 2020 Dec 9;10(12):503. doi: 10.3390/metabo10120503 (PMC7763243; doi:10.3390/metabo10120503)
Supplement: Supplementary file 1 [file metabolites-10-00503-s001.zip › Supplementary Tables/Table S3 Muscle amino acids.docx]

Table S3 Effects of dietary protein sources on free amino acid levels in the gastrocnemius muscle

|  | Casein | Beef  Leg | Pork  Leg | Chicken  Leg | Chicken  Breast | ANOVA |
| --- | --- | --- | --- | --- | --- | --- |
| Proline | 0.39±0.02^a^ | 0.24±0.02^b^ | 0.24±0.02^b^ | 0.24±0.02^b^ | 0.24±0.02^b^ | <.0001 |
| Tyrosine | 0.27±0.01^a^ | 0.20±0.01^b^ | 0.21±0.01^b^ | 0.21±0.01^b^ | 0.20±0.01^b^ | <.0001 |
| Glycine | 2.34±0.14^b^ | 3.00±0.18^ab^ | 3.10±0.26^ab^ | 3.32±0.32^a^ | 3.40±0.21^a^ | <0.05 |
| Isoleucine | 0.10±0.00^a^ | 0.08±0.00^b^ | 0.08±0.01^b^ | 0.08±0.00^ab^ | 0.08±0.01^ab^ | <0.05 |
| Leucine | 0.22±0.01^a^ | 0.15±0.01^b^ | 0.16±0.01^b^ | 0.16±0.01^b^ | 0.15±0.01^b^ | <0.05 |
| Methionine | 0.15±0.00^a^ | 0.14±0.01^ab^ | 0.14±0.00^ab^ | 0.13±0.00^b^ | 0.13±0.00^b^ | <0.05 |
| Phenylalanine | 0.15±0.00^a^ | 0.13±0.00^b^ | 0.13±0.00^b^ | 0.13±0.00^b^ | 0.13±0.00^b^ | <0.05 |
| Valine | 0.28±0.02^a^ | 0.20±0.01^b^ | 0.21±0.01^b^ | 0.20±0.00^b^ | 0.21±0.02^b^ | <0.05 |
| 2-Aminoadipic acid | 0.01±0.00 | 0.01±0.00 | 0.01±0.00 | 0.01±0.00 | 0.01±0.00 | NS |
| 2-Aminobutyric acid | 0.01±0.00 | 0.01±0.00 | 0.01±0.00 | 0.01±0.00 | 0.01±0.00 | NS |
| 4-Aminobutyric acid | 0.02±0.00 | 0.02±0.00 | 0.02±0.00 | 0.02±0.00 | 0.02±0.00 | NS |
| Alanine | 2.98±0.08 | 2.76±0.19 | 2.81±0.09 | 2.82±0.14 | 2.84±0.15 | NS |
| Asparagine | 0.14±0.01 | 0.13±0.01 | 0.14±0.01 | 0.14±0.01 | 0.14±0.01 | NS |
| Aspartic acid | 0.19±0.01 | 0.17±0.02 | 0.18±0.02 | 0.14±0.01 | 0.15±0.02 | NS |
| Cystine | 0.03±0.00 | 0.03±0.00 | 0.03±0.00 | 0.03±0.00 | 0.03±0.00 | NS |
| Glutamic acid | 0.77±0.09 | 0.81±0.08 | 0.85±0.06 | 0.75±0.04 | 0.81±0.05 | NS |
| Glutamine | 1.72±0.12 | 1.75±0.09 | 1.73±0.07 | 1.80±0.12 | 1.84±0.10 | NS |
| Histidine | 0.25±0.01 | 0.25±0.01 | 0.26±0.01 | 0.25±0.01 | 0.26±0.01 | NS |
| Hydroxyproline | 1.75±0.22 | 1.88±0.21 | 2.18±0.25 | 1.62±0.11 | 1.96±0.16 | NS |
| Lysine | 1.34±0.11 | 1.20±0.06 | 1.32±0.15 | 1.55±0.12 | 1.43±0.15 | NS |
| Ornithine | 0.19±0.00 | 0.20±0.01 | 0.20±0.00 | 0.20±0.00 | 0.20±0.01 | NS |
| Serine | 0.40±0.02 | 0.39±0.04 | 0.39±0.04 | 0.40±0.04 | 0.40±0.05 | NS |
| Threonine | 0.48±0.02 | 0.45±0.04 | 0.44±0.02 | 0.45±0.02 | 0.44±0.03 | NS |
| Tryptophan | 0.02±0.00 | 0.01±0.00 | 0.02±0.00 | 0.01±0.00 | 0.01±0.00 | NS |

Values (µmol/g) are means with their standard errors (n = 6). NS: not significant (P ≥ 0.05); ANOVA: analysis of variance. Different letters in the same line denote significantly different mean values according to the Tukey test (P < 0.05).
