# Supplementary material for: Effects of Dietary Defatted Meat Species on Metabolomic Profiles of Murine Liver, Gastrocnemius Muscle, and Cecal Content
Source: Metabolites. 2020 Dec 9;10(12):503. doi: 10.3390/metabo10120503 (PMC7763243; doi:10.3390/metabo10120503)
Supplement: Supplementary file 1 [file metabolites-10-00503-s001.zip › Supplementary Tables/Table S4 Number and name of metabolic pathway.docx]

Table S4 Number and name of metabolic pathway

| **Metab. No.** | **Pathway Name** |
| --- | --- |
| 1 | Aminoacyl-tRNA biosynthesis |
| 2 | Glycine, serine, and threonine metabolism |
| 3 | Valine, leucine, and isoleucine biosynthesis |
| 4 | Valine, leucine, and isoleucine degradation |
| 5 | Cysteine and methionine metabolism |
| 6 | Propanoate metabolism |
| 7 | β-Alanine metabolism |
| 8 | Fructose and mannose metabolism |
| 9 | Glycerolipid metabolism |
| 10 | Alanine, aspartate, and glutamate metabolism |
| 11 | Arginine and proline metabolism |
| 12 | Butanoate metabolism |
| 13 | Glutamate metabolism |
| 14 | Glyoxylate and dicarboxylate metabolism |
| 15 | Histidine metabolism |
| 16 | Phenylalanine, tyrosine, and tryptophan biosynthesis |
| 17 | Phenylalanine metabolism |
| 18 | Amino sugar and nucleotide sugar metabolism |
| 19 | Galactose metabolism |
| 20 | Pantothenate and CoA biosynthesis |
| 21 | Pentose phosphate pathway |
| 22 | Taurine and hypotaurine metabolism |

Metab. No., metabolic pathway number

Table S4 continued

|  | Casein | Beef  Leg | Pork  Leg | Chicken  Leg | Chicken Breast | ANOVA |
| --- | --- | --- | --- | --- | --- | --- |
| Lactitol | 97±9 | 102±11 | 91±8 | 108±9 | 102±8 | NS |
| Lauric acid | 104±8 | 102±11 | 94±10 | 97±10 | 103±11 | NS |
| Linoleic acid | 103±11 | 98±7 | 86±7 | 102±13 | 111±24 | NS |
| Lyxose | 116±4 | 99±14 | 87±7 | 103±7 | 96±7 | NS |
| Malic acid | 104±15 | 75±14 | 107±24 | 111±16 | 104±10 | NS |
| Malonic acid | 106±4 | 96±5 | 100±8 | 99±7 | 99±5 | NS |
| Maltose | 99±6 | 101±7 | 94±4 | 105±5 | 100±7 | NS |
| Mannose | 104±5 | 105±13 | 87±4 | 107±3 | 97±6 | NS |
| Mannose 6-phosphate | 90±9 | 105±14 | 88±10 | 113±12 | 105±9 | NS |
| Margaric acid | 105±8 | 99±4 | 97±7 | 98±5 | 102±11 | NS |
| meso-Erythritol | 100±4 | 99±7 | 102±6 | 110±5 | 89±6 | NS |
| Methylsuccinic acid | 97±4 | 116±9 | 100±8 | 91±5 | 95±9 | NS |
| Myristic acid | 103±8 | 98±6 | 100±4 | 101±4 | 99±10 | NS |
| N6-Acetyllysine | 94±9 | 107±9 | 112±11 | 96±4 | 91±6 | NS |
| N-Acetylmannosamine | 104±10 | 108±11 | 98±22 | 92±10 | 98±12 | NS |
| N-Acetylserine | 109±8 | 98±10 | 99±3 | 99±7 | 95±6 | NS |
| N-Butyrylglycine | 99±10 | 114±12 | 107±25 | 92±12 | 88±7 | NS |
| Niacinamide | 100±2 | 103±3 | 95±2 | 104±5 | 98±6 | NS |
| Nonanoic acid | 108±9 | 111±14 | 76±5 | 100±8 | 105±13 | NS |
| Norepinephrine | 103±5 | 108±7 | 95±3 | 96±8 | 99±4 | NS |
| O-Acetylserine | 104±5 | 116±21 | 93±2 | 94±6 | 93±5 | NS |
| Octanoic acid | 100±6 | 114±12 | 82±4 | 94±7 | 110±16 | NS |
| Octopamine | 111±8 | 106±10 | 92±5 | 96±6 | 95±7 | NS |
| Oleic acid | 109±9 | 99±7 | 89±8 | 99±9 | 104±16 | NS |
| O-Phosphoethanolamine | 102±4 | 102±7 | 100±5 | 98±4 | 97±9 | NS |
| Oxalacetic acid | 95±10 | 94±9 | 102±7 | 104±7 | 105±12 | NS |
| Palmitic acid | 106±7 | 99±3 | 97±4 | 102±4 | 96±7 | NS |
| Palmitoleic acid | 111±10 | 94±9 | 88±12 | 112±14 | 95±6 | NS |
| Pantothenic acid | 97±10 | 93±8 | 97±6 | 103±7 | 110±9 | NS |
| Phosphoenolpyruvic acid | 107±8 | 106±16 | 107±6 | 97±6 | 83±18 | NS |
| Phosphoric acid | 97±4 | 100±4 | 100±6 | 106±5 | 97±5 | NS |

Table S4 continued

|  | Casein | Beef  Leg | Pork  Leg | Chicken  Leg | Chicken Breast | ANOVA |
| --- | --- | --- | --- | --- | --- | --- |
| Pyrogallol | 105±7 | 96±4 | 94±4 | 104±4 | 101±9 | NS |
| Quinolinic acid | 125±19 | 105±11 | 88±4 | 98±6 | 84±7 | NS |
| Ribonic acid | 88±10 | 92±9 | 105±8 | 110±13 | 105±11 | NS |
| Ribose | 110±15 | 109±20 | 79±6 | 97±7 | 105±11 | NS |
| Ribose 5-phosphate | 110±12 | 116±8 | 88±5 | 94±9 | 91±12 | NS |
| Ribulose 5-phosphate | 109±11 | 108±11 | 87±3 | 97±7 | 99±10 | NS |
| Sedoheptulose 7-phosphate | 98±8 | 104±10 | 81±3 | 115±7 | 102±11 | NS |
| Spermidine | 105±9 | 97±9 | 95±3 | 101±6 | 102±7 | NS |
| Stearic acid | 102±5 | 100±6 | 98±5 | 103±4 | 98±8 | NS |
| Sucrose | 81±12 | 163±98 | 82±9 | 103±39 | 71±13 | NS |
| Taurine | 95± 6 | 94± 5 | 105± 3 | 110± 4 | 97± 7 | NS |
| Threonic acid | 97±7 | 95±7 | 105±12 | 105±19 | 98±11 | NS |
| Thymine | 98±4 | 98±3 | 102±4 | 108±6 | 94±4 | NS |
| Trehalose | 98±6 | 101±7 | 94±4 | 105±5 | 101±7 | NS |
| Triethanolamine | 91±4 | 90±10 | 121±13 | 109±17 | 90±11 | NS |
| Tyramine | 117±7 | 88±4 | 102±6 | 100±8 | 93±8 | NS |
| Uracil | 92±10 | 97±10 | 100±8 | 107±11 | 104±15 | NS |
| Urea | 97±6 | 101±7 | 101±8 | 111±7 | 89±5 | NS |
| Uridine | 107±13 | 96±8 | 94±6 | 104±12 | 100±8 | NS |
| Uridine monophosphate | 100±9 | 110±10 | 100±6 | 96±6 | 94±12 | NS |
| Xanthine | 100±8 | 99±8 | 95±6 | 106±6 | 101±9 | NS |
| Xanthosine | 101±11 | 105±18 | 83±13 | 105±16 | 106±21 | NS |
| Xanthosine monophosphate | 100±6 | 104±2 | 102±4 | 98±4 | 96±8 | NS |

Relative values are means with their standard errors (n = 6). NS: not significant (P ≥ 0.05); ANOVA: analysis of variance. Different letters in the same line denote significantly different mean values according to the Tukey test (P < 0.05).
