# Supplementary material for: Effects of Dietary Defatted Meat Species on Metabolomic Profiles of Murine Liver, Gastrocnemius Muscle, and Cecal Content
Source: Metabolites. 2020 Dec 9;10(12):503. doi: 10.3390/metabo10120503 (PMC7763243; doi:10.3390/metabo10120503)
Supplement: Supplementary file 1 [file metabolites-10-00503-s001.zip › Supplementary Tables/Table S5 Liver metabolites.docx]

Table S4 Effects of dietary protein sources on all semi-quantified metabolite levels in the liver

|  | Casein | Beef  Leg | Pork  Leg | Chicken  Leg | Chicken  Breast | ANOVA |
| --- | --- | --- | --- | --- | --- | --- |
| 1,5-Anhydro-glucitol | 111±4^ab^ | 97±5^bc^ | 129±8^a^ | 87±4^c^ | 76±5^c^ | <.0001 |
| 2-Hydroxyisovaleric acid | 128±9^a^ | 80±3^c^ | 105±5^ab^ | 99±2^bc^ | 88±6^bc^ | <.0001 |
| Carnosine | 7±1^d^ | 125±21^b^ | 242±30^a^ | 39±5^cd^ | 86±19^bc^ | <.0001 |
| 2-Hydroxybutyric acid | 139± 14^a^ | 93±10^b^ | 92±10^b^ | 97±10^ab^ | 79±5^b^ | <0.05 |
| 3-Hydroxypropionic acid | 90±1^b^ | 103±3^ab^ | 107±3^a^ | 97±2^ab^ | 102±7^ab^ | <0.05 |
| 4-Hydroxyphenyllactic acid | 142±7^a^ | 102±16^b^ | 90±4^b^ | 92±7^b^ | 74±7^b^ | <0.05 |
| 4-Hydroxyproline | 98±4^b^ | 91±2^b^ | 104±4^ab^ | 120±7^a^ | 87±5^b^ | <0.05 |
| β-Alanine | 85±5^c^ | 88±3^bc^ | 117±4^a^ | 100±5^abc^ | 111±9^ab^ | <0.05 |
| Creatinine | 48±6^b^ | 100±8^ab^ | 134±21^a^ | 128±22^a^ | 90±17^ab^ | <0.05 |
| Dihydroxyacetone phosphate | 132± 19 | 132±21 | 69±2 | 72±13 | 95±13 | <0.05 |
| Dimethylglycine | 90±10^ab^ | 115±14^ab^ | 83±10^ab^ | 76±4^b^ | 136±24^a^ | <0.05 |
| Dopamine | 112±7^a^ | 93±5^ab^ | 98±5^ab^ | 109±5^ab^ | 88±4^b^ | <0.05 |
| Glutaric acid | 154±13^a^ | 90±9^b^ | 92±5^b^ | 91±14^b^ | 74±12^b^ | <0.05 |
| Glycerol 3-phosphate | 133±20 | 133±22 | 68±2 | 71±14 | 95±13 | <0.05 |
| Glyoxylic acid | 91±10^ab^ | 114±14^ab^ | 83±9^ab^ | 76±3^b^ | 136±25^a^ | <0.05 |
| Homocysteine | 118±7^a^ | 98±2^ab^ | 96±5^ab^ | 99±7^ab^ | 89±6^b^ | <0.05 |
| Inositol | 82±4^b^ | 96±4^b^ | 101±2^ab^ | 121±4^a^ | 101±8^ab^ | <0.05 |
| Maleic acid | 126±9^a^ | 84±5^b^ | 98±7^ab^ | 99±6^ab^ | 92±6^b^ | <0.05 |
| Nicotinic acid | 114±10 | 90±5 | 87±5 | 110±7 | 99±6 | <0.05 |
| Pyruvic acid | 127±13^a^ | 113±12^ab^ | 97±9^ab^ | 96±13^ab^ | 67±12^b^ | <0.05 |
| Saccharopine | 162±15^a^ | 77±11^b^ | 99±7^b^ | 94±16^b^ | 68±14^b^ | <0.05 |
| Sorbitol | 86±13^b^ | 86±14^b^ | 92±11^ab^ | 137±11^a^ | 98±9^ab^ | <0.05 |
| Succinic acid | 86±4^b^ | 99±3^ab^ | 104±3^a^ | 111±3^a^ | 100±5^ab^ | <0.05 |
| Tartaric acid | 61±8^c^ | 95±14^abc^ | 129±21^ab^ | 143±21^a^ | 72±7^bc^ | <0.05 |
| Xylitol | 95±4^ab^ | 93±6^b^ | 100±2^ab^ | 115±4^a^ | 96±7^ab^ | <0.05 |
| 1,6-Anhydroglucose | 90±6 | 97±3 | 101±4 | 114±6 | 98±7 | NS |
| 1-Hexadecanol | 115±11 | 97±12 | 98±9 | 99±9 | 91±10 | NS |
| 2-Aminoethanol | 82±9 | 91±9 | 106±10 | 115±7 | 106±6 | NS |
| 2-Aminoisobutyric acid | 97±3 | 102±3 | 100±3 | 104±3 | 98±5 | NS |
| 2-Aminooctanoic acid | 105±2 | 106±4 | 96±4 | 97±3 | 96±6 | NS |
| 2-Aminopimelic acid | 114±20 | 97±15 | 120±16 | 94±13 | 75±16 | NS |

Table S4 continued

|  | Casein | Beef  Leg | Pork  Leg | Chicken  Leg | Chicken  Breast | ANOVA |
| --- | --- | --- | --- | --- | --- | --- |
| 2'-Deoxyuridine | 108±14 | 96±8 | 93±7 | 104±13 | 98±7 | NS |
| 2-Hydroxyglutaric acid | 92±6 | 93±6 | 120±14 | 111±11 | 84±10 | NS |
| 2-Hydroxyisobutyric acid | 102±7 | 102±6 | 101±5 | 103±4 | 91±7 | NS |
| 2-Ketobutyric acid | 137±24 | 90±9 | 87±3 | 98±12 | 87±11 | NS |
| 2-Ketoglutaric acid | 133±16 | 93±14 | 95±14 | 102±26 | 77±11 | NS |
| 3-Aminoglutaric acid | 122±16 | 93±15 | 96±20 | 93±17 | 96±11 | NS |
| 3-Aminoisobutyric acid | 110±4 | 95±2 | 100±7 | 98±7 | 96±10 | NS |
| 3-Hydroxybutyric acid | 104±8 | 108±9 | 94±6 | 90±6 | 104±13 | NS |
| 3-Methyladipic acid | 85±9 | 117±19 | 109±17 | 89±9 | 100±21 | NS |
| 3-Phosphoglyceric acid | 98±11 | 105±19 | 109±5 | 102±10 | 87±9 | NS |
| 4-Hydroxybenzoic acid | 97±4 | 93±4 | 98±4 | 125±28 | 88±5 | NS |
| 4-Hydroxyphenylacetic acid | 132±25 | 83±8 | 98±12 | 102±9 | 85±10 | NS |
| 5-Aminovaleric acid | 109±12 | 78±11 | 74±17 | 118±35 | 121±28 | NS |
| 5-Methoxytryptamine | 109±8 | 112±8 | 103±10 | 85±8 | 91±10 | NS |
| 5-Oxoproline | 108±5 | 98±4 | 97±4 | 101±4 | 96±6 | NS |
| Aconitic acid | 115±16 | 105±10 | 109±15 | 95±12 | 76±14 | NS |
| Adenine | 103±4 | 104±4 | 99±4 | 98±4 | 96±5 | NS |
| Adenosine | 96±3 | 98±4 | 104±5 | 105±5 | 97±7 | NS |
| Adenosine monophosphate | 101±6 | 104±4 | 101±6 | 97±5 | 96±10 | NS |
| Adipic acid | 86±9 | 101±8 | 108±5 | 100±5 | 105±18 | NS |
| Arabinose | 106±6 | 100±12 | 87±8 | 115±16 | 92±6 | NS |
| Arachidonic acid | 105±6 | 100±4 | 95±4 | 110±13 | 91±4 | NS |
| Arginine | 103±8 | 95±5 | 107±7 | 104±4 | 91±3 | NS |
| Ascorbic acid | 90±11 | 98±14 | 116±12 | 97±8 | 98±14 | NS |
| Azelaic acid | 93±10 | 86±6 | 112±5 | 98±12 | 111±20 | NS |
| Benzoic acid | 100±8 | 109±12 | 85±6 | 91±8 | 115±14 | NS |
| Cadaverine | 88±9 | 104±14 | 119±23 | 81±10 | 108±28 | NS |
| Caproic acid | 99±4 | 101±6 | 94±8 | 99±14 | 106±6 | NS |
| Catechol | 95±9 | 94±6 | 99±5 | 110±4 | 102±8 | NS |
| Cholesterol | 111±21 | 99±13 | 91±12 | 108±14 | 90±9 | NS |
| Citric acid | 113±19 | 97±15 | 120±16 | 95±14 | 75±16 | NS |

Table S4 continued

|  | Casein | Beef  Leg | Pork  Leg | Chicken  Leg | Chicken  Breast | ANOVA |
| --- | --- | --- | --- | --- | --- | --- |
| Cysteine | 109±9 | 106±11 | 90±5 | 99±7 | 97±10 | NS |
| Cytosine | 96±4 | 97±3 | 101±4 | 107±3 | 98±7 | NS |
| Decanoic acid | 106±4 | 105±6 | 92±4 | 97±5 | 100±7 | NS |
| Dihydrouracil | 101±8 | 94±9 | 93±5 | 105±12 | 107±13 | NS |
| Dihydroxyacetone | 124±26 | 106±31 | 68±9 | 90±16 | 111±18 | NS |
| Elaidic acid | 115±13 | 104±7 | 93±8 | 98±8 | 89±7 | NS |
| Fructose | 103±15 | 97±20 | 81±5 | 118±12 | 101±10 | NS |
| Fructose 1-phosphate | 114±10 | 101±6 | 96±5 | 100±7 | 88±13 | NS |
| Fructose 6-phosphate | 95±6 | 103±12 | 91±6 | 115±12 | 96±11 | NS |
| Fumaric acid | 105±15 | 75±12 | 108±22 | 107±14 | 104±10 | NS |
| Galacturonic acid | 99±5 | 105±5 | 94±5 | 101±12 | 100±6 | NS |
| Glucaric acid | 93±5 | 105±7 | 105±5 | 101±9 | 97±7 | NS |
| Gluconic acid | 92±5 | 105±7 | 106±5 | 100±9 | 97±7 | NS |
| Glucose | 101±2 | 104±4 | 99±5 | 100±2 | 95±5 | NS |
| Glucose 6-phosphate | 98±6 | 108±14 | 84±4 | 114±11 | 96±11 | NS |
| Glucuronic acid | 96±4 | 101±4 | 101±7 | 102±4 | 100±3 | NS |
| Glyceraldehyde 3-phosphate | 115±14 | 103±16 | 91±10 | 93±7 | 98±11 | NS |
| Glycolic acid | 102±1 | 99±3 | 101±5 | 102±3 | 97±5 | NS |
| Guanine | 97±6 | 103±5 | 100±4 | 100±2 | 99±6 | NS |
| Guanosine | 101±8 | 99±8 | 90±3 | 102±8 | 107±9 | NS |
| Hippuric acid | 92±14 | 94±15 | 92±7 | 115±10 | 107±13 | NS |
| Homoserine | 113±6 | 108±7 | 103±8 | 82±7 | 95±11 | NS |
| Hydroxylamine | 110±4 | 99±3 | 99±2 | 100±3 | 93±6 | NS |
| Hypotaurine | 89±9 | 103±14 | 120±23 | 80±10 | 108±28 | NS |
| Hypoxanthine | 99±11 | 95±10 | 94±7 | 105±10 | 108±12 | NS |
| Inosine | 95±12 | 97±12 | 86±5 | 109±13 | 113±16 | NS |
| Inosine monophosphate | 115±13 | 100±8 | 91±10 | 98±13 | 96±17 | NS |
| Isocitric acid | 93±9 | 89±9 | 114±7 | 107±9 | 97±3 | NS |
| Isovalerylglycine | 96±7 | 99±9 | 107±14 | 114±12 | 85±11 | NS |
| Kynurenine | 126±21 | 86±9 | 82±7 | 98±13 | 108±21 | NS |
| Lactic acid | 102±6 | 100±6 | 101±6 | 103±4 | 94±6 | NS |

Table S4 continued

|  | Casein | Beef  Leg | Pork  Leg | Chicken  Leg | Chicken  Breast | ANOVA |
| --- | --- | --- | --- | --- | --- | --- |
| Lactitol | 97±9 | 102±11 | 91±8 | 108±9 | 102±8 | NS |
| Lauric acid | 104±8 | 102±11 | 94±10 | 97±10 | 103±11 | NS |
| Linoleic acid | 103±11 | 98±7 | 86±7 | 102±13 | 111±24 | NS |
| Lyxose | 116±4 | 99±14 | 87±7 | 103±7 | 96±7 | NS |
| Malic acid | 104±15 | 75±14 | 107±24 | 111±16 | 104±10 | NS |
| Malonic acid | 106±4 | 96±5 | 100±8 | 99±7 | 99±5 | NS |
| Maltose | 99±6 | 101±7 | 94±4 | 105±5 | 100±7 | NS |
| Mannose | 104±5 | 105±13 | 87±4 | 107±3 | 97±6 | NS |
| Mannose 6-phosphate | 90±9 | 105±14 | 88±10 | 113±12 | 105±9 | NS |
| Margaric acid | 105±8 | 99±4 | 97±7 | 98±5 | 102±11 | NS |
| meso-Erythritol | 100±4 | 99±7 | 102±6 | 110±5 | 89±6 | NS |
| Methylsuccinic acid | 97±4 | 116±9 | 100±8 | 91±5 | 95±9 | NS |
| Myristic acid | 103±8 | 98±6 | 100±4 | 101±4 | 99±10 | NS |
| N6-Acetyllysine | 94±9 | 107±9 | 112±11 | 96±4 | 91±6 | NS |
| N-Acetylmannosamine | 104±10 | 108±11 | 98±22 | 92±10 | 98±12 | NS |
| N-Acetylserine | 109±8 | 98±10 | 99±3 | 99±7 | 95±6 | NS |
| N-Butyrylglycine | 99±10 | 114±12 | 107±25 | 92±12 | 88±7 | NS |
| Niacinamide | 100±2 | 103±3 | 95±2 | 104±5 | 98±6 | NS |
| Nonanoic acid | 108±9 | 111±14 | 76±5 | 100±8 | 105±13 | NS |
| Norepinephrine | 103±5 | 108±7 | 95±3 | 96±8 | 99±4 | NS |
| O-Acetylserine | 104±5 | 116±21 | 93±2 | 94±6 | 93±5 | NS |
| Octanoic acid | 100±6 | 114±12 | 82±4 | 94±7 | 110±16 | NS |
| Octopamine | 111±8 | 106±10 | 92±5 | 96±6 | 95±7 | NS |
| Oleic acid | 109±9 | 99±7 | 89±8 | 99±9 | 104±16 | NS |
| O-Phosphoethanolamine | 102±4 | 102±7 | 100±5 | 98±4 | 97±9 | NS |
| Oxalacetic acid | 95±10 | 94±9 | 102±7 | 104±7 | 105±12 | NS |
| Palmitic acid | 106±7 | 99±3 | 97±4 | 102±4 | 96±7 | NS |
| Palmitoleic acid | 111±10 | 94±9 | 88±12 | 112±14 | 95±6 | NS |
| Pantothenic acid | 97±10 | 93±8 | 97±6 | 103±7 | 110±9 | NS |
| Phosphoenolpyruvic acid | 107±8 | 106±16 | 107±6 | 97±6 | 83±18 | NS |
| Phosphoric acid | 97±4 | 100±4 | 100±6 | 106±5 | 97±5 | NS |

Table S4 continued

|  | Casein | Beef  Leg | Pork  Leg | Chicken  Leg | Chicken  Breast | ANOVA |
| --- | --- | --- | --- | --- | --- | --- |
| Pyrogallol | 105±7 | 96±4 | 94±4 | 104±4 | 101±9 | NS |
| Quinolinic acid | 125±19 | 105±11 | 88±4 | 98±6 | 84±7 | NS |
| Ribonic acid | 88±10 | 92±9 | 105±8 | 110±13 | 105±11 | NS |
| Ribose | 110±15 | 109±20 | 79±6 | 97±7 | 105±11 | NS |
| Ribose 5-phosphate | 110±12 | 116±8 | 88±5 | 94±9 | 91±12 | NS |
| Ribulose 5-phosphate | 109±11 | 108±11 | 87±3 | 97±7 | 99±10 | NS |
| Sedoheptulose 7-phosphate | 98±8 | 104±10 | 81±3 | 115±7 | 102±11 | NS |
| Spermidine | 105±9 | 97±9 | 95±3 | 101±6 | 102±7 | NS |
| Stearic acid | 102±5 | 100±6 | 98±5 | 103±4 | 98±8 | NS |
| Sucrose | 81±12 | 163±98 | 82±9 | 103±39 | 71±13 | NS |
| Taurine | 95± 6 | 94± 5 | 105± 3 | 110± 4 | 97± 7 | NS |
| Threonic acid | 97±7 | 95±7 | 105±12 | 105±19 | 98±11 | NS |
| Thymine | 98±4 | 98±3 | 102±4 | 108±6 | 94±4 | NS |
| Trehalose | 98±6 | 101±7 | 94±4 | 105±5 | 101±7 | NS |
| Triethanolamine | 91±4 | 90±10 | 121±13 | 109±17 | 90±11 | NS |
| Tyramine | 117±7 | 88±4 | 102±6 | 100±8 | 93±8 | NS |
| Uracil | 92±10 | 97±10 | 100±8 | 107±11 | 104±15 | NS |
| Urea | 97±6 | 101±7 | 101±8 | 111±7 | 89±5 | NS |
| Uridine | 107±13 | 96±8 | 94±6 | 104±12 | 100±8 | NS |
| Uridine monophosphate | 100±9 | 110±10 | 100±6 | 96±6 | 94±12 | NS |
| Xanthine | 100±8 | 99±8 | 95±6 | 106±6 | 101±9 | NS |
| Xanthosine | 101±11 | 105±18 | 83±13 | 105±16 | 106±21 | NS |
| Xanthosine monophosphate | 100±6 | 104±2 | 102±4 | 98±4 | 96±8 | NS |

Relative values are means with their standard errors (n = 6). NS: not significant (P ≥ 0.05); ANOVA: analysis of variance. Different letters in the same line denote significantly different mean values according to the Tukey test (P < 0.05).
