# Supplementary material for: Effects of Dietary Defatted Meat Species on Metabolomic Profiles of Murine Liver, Gastrocnemius Muscle, and Cecal Content
Source: Metabolites. 2020 Dec 9;10(12):503. doi: 10.3390/metabo10120503 (PMC7763243; doi:10.3390/metabo10120503)
Supplement: Supplementary file 1 [file metabolites-10-00503-s001.zip › Supplementary Tables/Table S6 Muscle metabolites.docx]

Table S5 Effect of dietary protein sources on all semi-quantified metabolite levels in the gastrocnemius muscle

|  | Casein | Beef  Leg | Pork  Leg | Chicken  Leg | Chicken  Breast | ANOVA |
| --- | --- | --- | --- | --- | --- | --- |
| 1,5-Anhydro-glucitol | 110±9^ab^ | 94±2^b^ | 123±5^a^ | 88±5^b^ | 86±7^b^ | <0.05 |
| 2-Hydroxybutyric acid | 129±12^a^ | 93±4^ab^ | 97±7^ab^ | 92±5^ab^ | 88±13^b^ | <0.05 |
| 2-Hydroxyisovaleric acid | 120±15^a^ | 82±6^b^ | 98±4^ab^ | 101±3^ab^ | 99±5^ab^ | <0.05 |
| 3-Hydroxyisobutyric acid | 144±13^a^ | 92±7^b^ | 87±8^b^ | 89±10^b^ | 88±8^b^ | <0.05 |
| Inositol | 83±9^b^ | 104±4^ab^ | 97±4^b^ | 123±6^a^ | 93±3^b^ | <0.05 |
| Palmitoleic acid | 110±10^a^ | 102±7^ab^ | 100±4^ab^ | 106±6^ab^ | 81±5^b^ | <0.05 |
| Sarcosine | 78±3^b^ | 113±8^a^ | 105±7^a^ | 101±6^ab^ | 104±7^ab^ | <0.05 |
| 1-Hexadecanol | 95±5 | 94±2 | 104±6 | 99±5 | 108±5 | NS |
| 2,3-Bisphosphoglyceric acid | 87±3 | 88±5 | 103±18 | 114±12 | 109±19 | NS |
| 2-Aminoethanol | 87±18 | 107±11 | 105±16 | 92±8 | 108±15 | NS |
| 2-Aminooctanoic acid | 101±9 | 104±6 | 99±5 | 97±3 | 100±4 | NS |
| 2-Aminopimelic acid | 85±10 | 109±8 | 108±18 | 89±8 | 109±15 | NS |
| 2-Deoxy-glucose | 90±9 | 98±8 | 92±18 | 119±22 | 101±11 | NS |
| 2-Hydroxyglutaric acid | 78±7 | 106±13 | 107±7 | 100±4 | 109±7 | NS |
| 2-Ketoisocaproic acid | 108±16 | 87±8 | 90±13 | 100±11 | 116±19 | NS |
| 2-Phosphoglyceric acid | 50±24 | 68±35 | 97±53 | 174±97 | 111±47 | NS |
| 3-Aminoglutaric acid | 103±10 | 109±5 | 108±8 | 89±5 | 90±7 | NS |
| 3-Hydroxybutyric acid | 106±3 | 111±12 | 86±7 | 91±8 | 106±14 | NS |
| 3-Hydroxyglutaric acid | 100±1 | 99±1 | 100±1 | 101±1 | 100±1 | NS |
| 3-Hydroxyisovaleric acid | 105±4 | 96±2 | 101±3 | 98±3 | 99±4 | NS |
| 3-Hydroxypropionic acid | 101±3 | 96±3 | 102±4 | 101±3 | 100±5 | NS |
| 3-Methoxy-4-hydroxybenzoic acid | 109±17 | 86±3 | 104±11 | 92±8 | 109±16 | NS |
| 3-Phenyllactic acid | 100±3 | 100±4 | 101±3 | 99±2 | 100±3 | NS |
| 3-Phosphoglyceric acid | 45±26 | 58±32 | 95±58 | 189±121 | 112±53 | NS |
| 4-Hydroxybenzoic acid | 104±18 | 104±18 | 108±23 | 92±6 | 92±14 | NS |
| 4-Hydroxyphenylacetic acid | 102±27 | 79±19 | 98±28 | 103±20 | 118±38 | NS |
| 4-Hydroxyphenyllactic acid | 121±13 | 83±11 | 90±14 | 102±10 | 104±18 | NS |
| 5-Aminovaleric acid | 93±18 | 88±15 | 83±17 | 102±12 | 134±38 | NS |
| 5-Oxoproline | 96±10 | 102±7 | 103±4 | 101±3 | 99±6 | NS |
| β-Alanine | 100±10 | 96±10 | 104±10 | 98± 6 | 102±10 | NS |

Table S5 continued

|  | Casein | Beef  Leg | Pork  Leg | Chicken  Leg | Chicken  Breast | ANOVA |
| --- | --- | --- | --- | --- | --- | --- |
| Acetylglycine | 113±19 | 101±10 | 93±8 | 95±3 | 98±11 | NS |
| Aconitic acid | 93±11 | 105±11 | 107±19 | 90±6 | 106±14 | NS |
| Adenine | 95±4 | 96±2 | 102±3 | 104±3 | 103±4 | NS |
| Adenosine | 95±12 | 101±7 | 104±8 | 101±4 | 99±7 | NS |
| Adenosine monophosphate | 95±9 | 98±5 | 102±9 | 107±7 | 98±8 | NS |
| Arabinose | 96±13 | 96±9 | 106±12 | 97±7 | 106±13 | NS |
| Arginine | 85±6 | 95±1 | 102±13 | 120±10 | 98±8 | NS |
| Ascorbic acid | 79±9 | 108±16 | 112±13 | 98±8 | 103±18 | NS |
| Azelaic acid | 90±17 | 103±32 | 121±63 | 102±33 | 85±9 | NS |
| Batyl alcohol | 97±4 | 98±4 | 101±4 | 101±6 | 102±4 | NS |
| Benzoic acid | 106±10 | 92±5 | 98±10 | 105±12 | 99±12 | NS |
| Cadaverine | 95±5 | 115±13 | 100±4 | 82±3 | 108±10 | NS |
| Caproic acid | 102±9 | 98±8 | 97±9 | 104±8 | 99±11 | NS |
| Carnosine | 119±21 | 81±17 | 86±14 | 105±22 | 108±26 | NS |
| Catechol | 101±8 | 98±7 | 97±6 | 100±4 | 104±3 | NS |
| Cholesterol | 85±16 | 122±17 | 103±16 | 109±21 | 80±9 | NS |
| Citramalic acid | 98±6 | 98±8 | 101±8 | 100±3 | 103±8 | NS |
| Citric acid | 85±11 | 109±8 | 109±19 | 89±9 | 109±16 | NS |
| Creatinine | 106±12 | 97±15 | 97±13 | 104±14 | 96±10 | NS |
| Cystamine | 102±9 | 89±3 | 101±10 | 110±10 | 98±8 | NS |
| Cysteine | 106±8 | 96±6 | 103±10 | 94±4 | 101±14 | NS |
| Cytosine | 100±8 | 97±3 | 101±3 | 101±2 | 102±4 | NS |
| Decanoic acid | 106±12 | 97±7 | 96±4 | 105±9 | 96±7 | NS |
| Dihydrouracil | 95±17 | 92±12 | 101±18 | 101±9 | 111±18 | NS |
| Dihydroxyacetone phosphate | 100±9 | 103±6 | 103±11 | 97±13 | 97±7 | NS |
| Dimethylglycine | 92±11 | 104±10 | 101±10 | 97±5 | 107±9 | NS |
| Elaidic acid | 106±9 | 100±6 | 100±4 | 99±5 | 95±3 | NS |
| Fructose | 104±26 | 91±22 | 96±22 | 94±18 | 116±31 | NS |
| Fructose 1-phosphate | 28±3 | 23±3 | 67±47 | 139±112 | 244±223 | NS |
| Fucose | 85±20 | 91±20 | 104±31 | 101±17 | 119±31 | NS |
| Fumaric acid | 98±7 | 105±10 | 101±6 | 99±3 | 96±4 | NS |

Table S5 continued

|  | Casein | Beef  Leg | Pork  Leg | Chicken  Leg | Chicken  Breast | ANOVA |
| --- | --- | --- | --- | --- | --- | --- |
| Galactosamine | 96±8 | 93±5 | 93±8 | 116±6 | 102±11 | NS |
| Galactose | 95±5 | 101±2 | 105±4 | 101±6 | 98±3 | NS |
| Galacturonic acid | 88±22 | 90±21 | 98±28 | 107±16 | 118±31 | NS |
| Glucaric acid | 90±17 | 93±15 | 101±18 | 107±10 | 110±21 | NS |
| Gluconic acid | 107±9 | 96±2 | 100±5 | 97±4 | 100±6 | NS |
| Glucose | 96±3 | 100±2 | 104±3 | 101±5 | 99±3 | NS |
| Glucuronic acid | 88±22 | 90±21 | 97±28 | 106±16 | 119±31 | NS |
| Glutaric acid | 97±9 | 99±11 | 107±18 | 100±10 | 97±7 | NS |
| Glyceric acid | 102±15 | 93±5 | 98±7 | 107±13 | 100±9 | NS |
| Glycerol | 100±8 | 104±7 | 98±5 | 100±6 | 97±3 | NS |
| Glycerol 3-phosphate | 99±10 | 104±7 | 102±12 | 97±12 | 97±6 | NS |
| Glycolic acid | 91±10 | 96±4 | 101±6 | 109±6 | 102±7 | NS |
| Guanine | 101±8 | 95±4 | 105±5 | 100±4 | 99±6 | NS |
| Guanosine | 97±20 | 105±11 | 96±7 | 100±8 | 102±4 | NS |
| Homocysteine | 100±16 | 100±9 | 101±8 | 98±6 | 100±11 | NS |
| Homoserine | 97±6 | 100±6 | 99±5 | 98±4 | 106±5 | NS |
| Hydroquinone | 98±8 | 96±5 | 98±4 | 101±4 | 107±5 | NS |
| Hydroxylamine | 100±6 | 93±6 | 95±3 | 104±6 | 108±8 | NS |
| Hypotaurine | 94±6 | 115±13 | 100±5 | 82±3 | 108±9 | NS |
| Hypoxanthine | 100±5 | 99±3 | 99±3 | 100±2 | 102±3 | NS |
| Inosine | 97±12 | 102±5 | 95±3 | 99±6 | 107±7 | NS |
| Isocitric acid | 97±13 | 112±8 | 103±10 | 85±8 | 103±16 | NS |
| Lactic acid | 99±2 | 99±2 | 100±3 | 101±4 | 100±3 | NS |
| Lactitol | 98±15 | 115±21 | 106±22 | 97±15 | 84±9 | NS |
| Lauric acid | 102±5 | 98±2 | 99±3 | 104±4 | 97±5 | NS |
| Linoleic acid | 92±5 | 109±7 | 103±10 | 107±10 | 90±3 | NS |
| Maleic acid | 100±6 | 98±4 | 101±6 | 101±4 | 100±5 | NS |
| Malic acid | 101±8 | 100±6 | 101±4 | 100±3 | 98±4 | NS |
| Maltose | 97±14 | 115±21 | 107±23 | 98±16 | 83±9 | NS |
| Mannose 6-phosphate | 15±4 | 14±7 | 60±52 | 165±132 | 247±238 | NS |
| Margaric acid | 98±4 | 96±3 | 107±3 | 98±4 | 101±4 | NS |

Table S5 continued

|  | Casein | Beef  Leg | Pork  Leg | Chicken  Leg | Chicken  Breast | ANOVA |
| --- | --- | --- | --- | --- | --- | --- |
| meso-Erythritol | 89±19 | 92±17 | 99±21 | 99±9 | 121±27 | NS |
| Methylsuccinic acid | 90±9 | 107±11 | 100±11 | 97±7 | 106±13 | NS |
| Monostearin | 100±3 | 96±2 | 101±3 | 101±3 | 102±3 | NS |
| Myristic acid | 98±3 | 96±2 | 108±4 | 99±2 | 99±3 | NS |
| N-Acetylglutamine | 99±7 | 90±2 | 98±9 | 111±10 | 102±8 | NS |
| N-Acetylserine | 94±12 | 94±8 | 101±15 | 101±6 | 109±16 | NS |
| Niacinamide | 98±7 | 103±3 | 101±3 | 100±3 | 98±2 | NS |
| Nicotinic acid | 111±9 | 97±10 | 95±8 | 106±11 | 91±3 | NS |
| Nonanoic acid | 104±11 | 97±3 | 96±4 | 108±16 | 94±5 | NS |
| Norepinephrine | 77±9 | 168±88 | 83±9 | 84±8 | 88±11 | NS |
| Octanoic acid | 97±6 | 96±2 | 96±4 | 106±8 | 105±13 | NS |
| Octopamine | 99±14 | 102±7 | 101±11 | 104±8 | 95±7 | NS |
| Oleic acid | 102±5 | 99±6 | 101±3 | 102±3 | 97±5 | NS |
| O-Phosphoethanolamine | 98±8 | 102±5 | 102±7 | 103±4 | 95±4 | NS |
| Oxalacetic acid | 101±4 | 98±7 | 100±5 | 101±6 | 100±5 | NS |
| Oxalic acid | 102±7 | 108±9 | 101±7 | 98±4 | 92±6 | NS |
| Palmitic acid | 97±3 | 97±3 | 107±3 | 98±4 | 101±3 | NS |
| Pantothenic acid | 90±8 | 95±6 | 98±6 | 112±4 | 105±5 | NS |
| Phenylacetic acid | 100±6 | 100±7 | 98±5 | 100±5 | 101±5 | NS |
| Protocatechuic acid | 84±8 | 156±84 | 80±6 | 76±7 | 104±15 | NS |
| Putrescine | 92±18 | 83±12 | 94±19 | 112±13 | 120±25 | NS |
| Pyrogallol | 102±14 | 104±12 | 99±7 | 95±4 | 100±4 | NS |
| Pyruvic acid | 118±12 | 93±7 | 96±12 | 100±5 | 93±11 | NS |
| Rhamnose | 87±19 | 90±19 | 104±29 | 103±16 | 116±28 | NS |
| Ribonic acid | 95±12 | 98±9 | 98±11 | 103±6 | 106±15 | NS |
| Stearic acid | 98±4 | 97±3 | 106±3 | 98±3 | 101±3 | NS |
| Succinic acid | 97±29 | 119±52 | 99±13 | 89±13 | 96±14 | NS |
| Sucrose | 91±26 | 100±47 | 84±16 | 158±73 | 67±14 | NS |
| Tartaric acid | 70±22 | 79±22 | 104±39 | 115±19 | 131±41 | NS |
| Taurine | 99± 3 | 99± 1 | 101± 2 | 102± 4 | 100± 1 | NS |
| Threitol | 89±20 | 91±18 | 100±23 | 100±11 | 120±27 | NS |

Table S5 continued

|  | Casein | Beef  Leg | Pork  Leg | Chicken  Leg | Chicken  Breast | ANOVA |
| --- | --- | --- | --- | --- | --- | --- |
| Threonic acid | 114±7 | 91±6 | 95±6 | 109±13 | 91±11 | NS |
| Thymine | 100±10 | 97±4 | 100±8 | 100±6 | 104±10 | NS |
| Trehalose | 99±15 | 115±21 | 105±23 | 97±16 | 84±9 | NS |
| Triethanolamine | 110±14 | 86±4 | 91±9 | 104±8 | 110±20 | NS |
| Tyramine | 101±7 | 89±1 | 97±11 | 112±11 | 101±8 | NS |
| Uracil | 97±9 | 98±6 | 100±5 | 101±4 | 104±4 | NS |
| Urea | 92±17 | 92±13 | 99±17 | 104±9 | 114±23 | NS |
| Uric acid | 94±16 | 99±12 | 98±15 | 99±10 | 109±20 | NS |
| Xanthine | 100±10 | 103±5 | 98±5 | 101±6 | 99±6 | NS |
| Xanthosine monophosphate | 95±8 | 98±5 | 102±8 | 107±7 | 98±8 | NS |

Relative values are means with their standard errors (n = 6). NS: not significant (P ≥ 0.05); ANOVA: analysis of variance. Different letters in the same line denote significantly different mean values according to the Tukey test (P < 0.05).
