# Supplementary material for: Effects of Dietary Defatted Meat Species on Metabolomic Profiles of Murine Liver, Gastrocnemius Muscle, and Cecal Content
Source: Metabolites. 2020 Dec 9;10(12):503. doi: 10.3390/metabo10120503 (PMC7763243; doi:10.3390/metabo10120503)
Supplement: Supplementary file 1 [file metabolites-10-00503-s001.zip › Supplementary Tables/Table S7 Cecal content metabolites.docx]

Table S6 Effect of dietary protein sources on all semi-quantified metabolite levels in the cecal content

|  | Casein | Beef  Leg | Pork  Leg | Chicken  Leg | Chicken  Breast | ANOVA |
| --- | --- | --- | --- | --- | --- | --- |
| 4-Hydroxyphenylacetic acid | 202±20^a^ | 58±10^b^ | 60±7^b^ | 102±27^b^ | 78±23^b^ | <.0001 |
| β-Alanine | 20±3^b^ | 268±30^a^ | 86±14^b^ | 42±4^b^ | 83±18^b^ | <.0001 |
| Carnosine | 0±0^b^ | 289±53^a^ | 180±33^a^ | 4±1^b^ | 27±11^b^ | <.0001 |
| N-Acetylglutamine | 32±7^c^ | 30±4^c^ | 150±32^ab^ | 200±13^a^ | 89±40^bc^ | <.0001 |
| Nicotinamide | 16±1^d^ | 106±6^b^ | 226±13^a^ | 86±5^bc^ | 65±12^c^ | <.0001 |
| Phenylacetic acid | 42±5^b^ | 97±10^b^ | 100±18^b^ | 174±17^a^ | 86±16^b^ | <.0001 |
| 2-Aminobutyric acid | 114±12^ab^ | 72±9^b^ | 65±13^b^ | 147±26^a^ | 103±23^ab^ | <0.05 |
| 2-Aminoethanol | 177±26^a^ | 92±27^b^ | 65±4^b^ | 73±5^b^ | 93±19^b^ | <0.05 |
| 2-Deoxy-glucose | 51±7^b^ | 99±14^ab^ | 115±16^a^ | 134±13^a^ | 101±19^ab^ | <0.05 |
| 2-Hydroxybutyric acid | 222±78^a^ | 77±17^ab^ | 47±6^b^ | 70±6^b^ | 84±15^ab^ | <0.05 |
| 2-Hydroxyisovaleric acid | 372±125^a^ | 36±12^b^ | 18±5^b^ | 21±5^b^ | 53±18^b^ | <0.05 |
| 2-Ketobutyric acid | 114±7^a^ | 92±3^b^ | 97±4^ab^ | 101±2^ab^ | 97±6^ab^ | <0.05 |
| 2-Ketoisocaproic acid | 78±6^b^ | 85±4^ab^ | 101±14^ab^ | 140±17^a^ | 97±21^ab^ | <0.05 |
| 3-Hydroxypropionic acid | 83±6^b^ | 127±8^a^ | 90±3^b^ | 104±9^ab^ | 97±9^ab^ | <0.05 |
| 3-Methyl-2-oxovaleric acid | 80±8^b^ | 82±3^b^ | 99±14^ab^ | 138±12^a^ | 101±21^ab^ | <0.05 |
| 3-Sulfinoalanine | 55±8^b^ | 88±14^b^ | 103±15^ab^ | 167±27^a^ | 87±14^b^ | <0.05 |
| 4-Hydroxyproline | 9±2^b^ | 83±12^ab^ | 90±26^ab^ | 221±75^a^ | 97±15^ab^ | <0.05 |
| Adenosine monophosphate | 253±90^a^ | 68±22^ab^ | 86±34^ab^ | 53±22^b^ | 40±14^b^ | <0.05 |
| Adenosine | 179±18^a^ | 59±9^b^ | 87±11^b^ | 78±25^b^ | 97±27^b^ | <0.05 |
| Ascorbic acid | 121±23 | 72±11 | 67±15 | 108±16 | 133±13 | <0.05 |
| Azelaic acid | 58±4^b^ | 105±12^a^ | 116±8^a^ | 111±5^a^ | 110±13^a^ | <0.05 |
| Cysteine | 57±10^b^ | 92±4^ab^ | 117±18^a^ | 138±16^a^ | 96±15^ab^ | <0.05 |
| Dihydroxyacetone phosphate | 74±13 | 117±18 | 126±12 | 63±13 | 120±19 | <0.05 |
| Fructose 6-phosphate | 64±13^b^ | 93±12^ab^ | 127±22^ab^ | 71±19^ab^ | 145±24^a^ | <0.05 |
| Galactose | 148±39^a^ | 48±7^b^ | 77±10^ab^ | 153±29^a^ | 74±8^ab^ | <0.05 |
| Glucose 6-phosphate | 63±14^b^ | 90±12^ab^ | 129±24^ab^ | 69±19^ab^ | 149±25^a^ | <0.05 |
| Glutaric acid | 85±16^b^ | 154±20^a^ | 104±10^ab^ | 83±5^b^ | 74±5^b^ | <0.05 |
| Glyceric acid | 154±29^a^ | 74±11^b^ | 99±8^ab^ | 96±7^ab^ | 77±17^b^ | <0.05 |
| Glycerol 3-phosphate | 74±13^ab^ | 116±17^ab^ | 129±14^a^ | 62±14^b^ | 118±18^ab^ | <0.05 |
| Glycine | 106±13^ab^ | 97±16^ab^ | 72±13^b^ | 137±13^a^ | 88±9^ab^ | <0.05 |
| Guanosine | 167±48^a^ | 72±9^ab^ | 64±5^b^ | 84±7^ab^ | 113±16^ab^ | <0.05 |
| Homocysteine | 134±16^a^ | 87±12^ab^ | 100±9^ab^ | 61±4^b^ | 117±15^a^ | <0.05 |

Table S6 continued

|  | Casein | Beef  Leg | Pork  Leg | Chicken  Leg | Chicken  Breast | ANOVA |
| --- | --- | --- | --- | --- | --- | --- |
| Isoleucine | 147±29 | 63±13 | 63±18 | 147±25 | 80±20 | <0.05 |
| Leucine | 133±21 | 73±13 | 70±14 | 136±14 | 89±16 | <0.05 |
| Mannose 6-phosphate | 64±14^b^ | 94±14^ab^ | 124±21^ab^ | 68±19^b^ | 151±26^a^ | <0.05 |
| Mannose | 132±24^a^ | 61±10^b^ | 75±9^ab^ | 120±15^ab^ | 112±19^ab^ | <0.05 |
| N-Acetylaspartic acid | 33±8^c^ | 56±11^bc^ | 142±28^ab^ | 163±22^a^ | 107±36^abc^ | <0.05 |
| Pantothenic acid | 120±18^ab^ | 77±8^b^ | 70±7^b^ | 137±18^a^ | 95±13^ab^ | <0.05 |
| Proline | 146±29^a^ | 66±8^b^ | 72±14^ab^ | 131±19^ab^ | 85±15^ab^ | <0.05 |
| Ribose | 102±10^ab^ | 74±8^b^ | 120±5^a^ | 109±8^ab^ | 95±13^ab^ | <0.05 |
| Ribulose 5-phosphate | 48±14 | 137±40 | 136±16 | 50±17 | 129±26 | <0.05 |
| Sedoheptulose 7-phosphate | 46±14^b^ | 112±24^ab^ | 143±24^a^ | 55±17^ab^ | 145±29^a^ | <0.05 |
| Serine | 131±16^a^ | 77±9^b^ | 84±10^ab^ | 124±10^ab^ | 84±13^ab^ | <0.05 |
| Tagatose | 150±25^a^ | 65±15^b^ | 69±6^b^ | 127±16^ab^ | 89±14^ab^ | <0.05 |
| Threitol | 153±22^a^ | 90±11^b^ | 83±9^b^ | 83±12^b^ | 92±13^b^ | <0.05 |
| Threonine | 137±28 | 69±8 | 78±17 | 131±17 | 85±16 | <0.05 |
| Valine | 143±25 | 69±13 | 67±16 | 138±18 | 82±18 | <0.05 |
| 2-Aminopimelic acid | 96±30 | 90±29 | 144±31 | 88±19 | 83±15 | NS |
| 2-Hydroxyglutaric acid | 124±27 | 104±17 | 99±19 | 66±9 | 106±11 | NS |
| 2-Hydroxyisobutyric acid | 106±18 | 91±10 | 102±12 | 77±5 | 125±22 | NS |
| 2-Ketoglutaric acid | 49±11 | 116±24 | 140±14 | 80±12 | 115±41 | NS |
| 2-Keto-isovaleric acid | 74±9 | 98±12 | 95±13 | 135±18 | 98±18 | NS |
| 3-Aminoglutaric acid | 90±9 | 66±13 | 148±24 | 119±20 | 76±29 | NS |
| 3-Aminoisobutyric acid | 135±17 | 139±53 | 69±16 | 80±10 | 77±11 | NS |
| 3-Hydroxybutyric acid | 118±14 | 89±3 | 109±5 | 86±9 | 97±9 | NS |
| 3-Hydroxyglutaric acid | 103±2 | 99±1 | 100±2 | 99±1 | 100±2 | NS |
| 3-Hydroxyisovaleric acid | 82±4 | 104±6 | 93±5 | 111±8 | 111±19 | NS |
| 3-Hydroxypyruvic acid | 84±17 | 93±16 | 122±24 | 105±11 | 96±10 | NS |
| 3-Methoxy-4-hydroxybenzoic acid | 96±22 | 74±35 | 161±35 | 72±20 | 97±24 | NS |
| 3-Phenyllactic acid | 145±26 | 79±16 | 89±7 | 86±9 | 101±17 | NS |
| 4-Aminobutyric acid | 65±8 | 113±13 | 84±12 | 93±15 | 145±58 | NS |
| 4-Hydroxybenzoic acid | 109±18 | 76±12 | 100±14 | 119±15 | 96±15 | NS |
| 5-Oxoproline | 115±14 | 79±8 | 107±15 | 104±9 | 95±17 | NS |

Table S6 continued

|  | Casein | Beef  Leg | Pork  Leg | Chicken  Leg | Chicken  Breast | ANOVA |
| --- | --- | --- | --- | --- | --- | --- |
| 7-Methylguanine | 89±10 | 88±8 | 104±9 | 107±13 | 112±24 | NS |
| Adenine | 128±27 | 77±11 | 100±10 | 100±8 | 95±13 | NS |
| Adipic acid | 63±11 | 103±34 | 124±39 | 73±12 | 137±32 | NS |
| Arabinose | 117±12 | 79±10 | 105±16 | 121±14 | 78±12 | NS |
| Arginine | 109±15 | 88±15 | 98±22 | 113±9 | 92±14 | NS |
| Asparagine | 113±22 | 78±17 | 67±17 | 155±39 | 87±22 | NS |
| Benzoic acid | 97±9 | 91±9 | 111±9 | 98±9 | 103±17 | NS |
| Cadaverine | 64±7 | 106±14 | 82±17 | 90±8 | 158±44 | NS |
| Caproic acid | 93±7 | 91±3 | 104±4 | 101±12 | 111±18 | NS |
| Catechol | 114±11 | 94±12 | 95±5 | 90±5 | 108±14 | NS |
| Citramalic acid | 104±20 | 98±16 | 89±12 | 112±16 | 97±13 | NS |
| Citric acid | 104±23 | 93±22 | 127±25 | 96±8 | 80±11 | NS |
| Cytosine | 163±49 | 78±9 | 90±11 | 84±6 | 84±6 | NS |
| Decanoic acid | 101±9 | 95±5 | 103±6 | 95±5 | 106±12 | NS |
| Dihydroxyacetone | 147±26 | 68±15 | 82±7 | 109±20 | 94±20 | NS |
| Dodecanedioic acid | 66±21 | 65±26 | 100±36 | 171±20 | 99±53 | NS |
| Fumaric acid | 125±21 | 102±19 | 118±7 | 62±11 | 93±20 | NS |
| Galacturonic acid | 145±70 | 71±7 | 97±16 | 112±25 | 75±7 | NS |
| Gluconic acid | 153±62 | 133±58 | 48±25 | 30±5 | 135±43 | NS |
| Glucosamine | 116±18 | 86±7 | 72±15 | 117±11 | 109±14 | NS |
| Glucose | 115±18 | 83±9 | 80±12 | 106±6 | 116±10 | NS |
| Glucuronic acid | 150±79 | 64±8 | 94±14 | 114±29 | 78±8 | NS |
| Glutaconic acid | 103±16 | 63±14 | 124±27 | 101±15 | 109±19 | NS |
| Glutamine | 121±19 | 89±15 | 76±17 | 118±20 | 96±21 | NS |
| Glycerol | 110±14 | 85±3 | 100±11 | 111±11 | 95±9 | NS |
| Glycolic acid | 90±13 | 92±4 | 102±9 | 118±9 | 97±10 | NS |
| Glycyl-Glycine | 67±7 | 103±17 | 80±18 | 93±7 | 156±43 | NS |
| Guanine | 144±33 | 77±5 | 81±6 | 90±8 | 108±14 | NS |
| Histidine | 87±20 | 115±18 | 86±29 | 122±10 | 90±18 | NS |
| Homoserine | 99±11 | 86±14 | 129±21 | 86±7 | 100±9 | NS |
| Hydroquinone | 111±15 | 104±9 | 101±5 | 90±6 | 94±6 | NS |
| Hydroxylamine | 108±8 | 95±4 | 103±5 | 95±4 | 98±5 | NS |

Table S6 continued

|  | Casein | Beef  Leg | Pork  Leg | Chicken  Leg | Chicken  Breast | ANOVA |
| --- | --- | --- | --- | --- | --- | --- |
| Hypoxanthine | 90±9 | 90±5 | 116±9 | 109±9 | 95±12 | NS |
| Inosine | 101±20 | 91±15 | 88±11 | 73±8 | 147±28 | NS |
| Inositol | 116±20 | 76±20 | 97±16 | 120±14 | 91±14 | NS |
| Lactic acid | 211±98 | 70±18 | 41±10 | 90±31 | 88±32 | NS |
| Lactitol | 189±92 | 50±9 | 33±16 | 53±12 | 175±77 | NS |
| Lauric acid | 107±6 | 94±4 | 103±5 | 94±3 | 101±7 | NS |
| Linoleic acid | 100±18 | 99±10 | 100±7 | 109±15 | 93±24 | NS |
| Lysine | 89±12 | 97±16 | 86±18 | 129±11 | 99±15 | NS |
| Maleic acid | 102±11 | 94±5 | 108±6 | 96±3 | 99±9 | NS |
| Malic acid | 125±22 | 113±16 | 98±11 | 60±12 | 103±21 | NS |
| Malonic acid | 97±9 | 111±13 | 90±14 | 110±13 | 92±13 | NS |
| Margaric acid | 105±9 | 95±4 | 106±6 | 96±3 | 97±7 | NS |
| Methionine | 124±18 | 75±11 | 77±16 | 137±23 | 87±17 | NS |
| Methylsuccinic acid | 84±9 | 108±12 | 115±15 | 106±9 | 86±13 | NS |
| Myristic acid | 105±8 | 93±5 | 108±7 | 95±3 | 99±5 | NS |
| N-Acetylmannosamine | 122±22 | 67±9 | 101±16 | 122±11 | 87±14 | NS |
| N-Acetylneuraminic acid | 208±124 | 70±11 | 73±6 | 81±14 | 67±15 | NS |
| N-Butyrylglycine | 108±11 | 98±2 | 101±4 | 96±7 | 97±7 | NS |
| Nicotinic acid | 87±7 | 95±4 | 111±5 | 97±6 | 110±10 | NS |
| Nonanoic acid | 98±6 | 89±8 | 112±11 | 93±11 | 108±17 | NS |
| Octanoic acid | 107±9 | 92±4 | 106±4 | 97±4 | 99±7 | NS |
| Oleic acid | 96±8 | 89±7 | 119±12 | 105±8 | 90±13 | NS |
| Ornithine | 113±27 | 83±19 | 95±23 | 116±11 | 94±25 | NS |
| Oxalic acid | 74±11 | 119±35 | 130±15 | 109±41 | 69±25 | NS |
| Palmitoleic acid | 116±15 | 87±6 | 104±11 | 90±2 | 102±11 | NS |
| Phenylalanine | 119±19 | 78±13 | 76±18 | 139±18 | 89±17 | NS |
| Putrescine | 109±13 | 92±6 | 95±5 | 101±6 | 103±6 | NS |
| Pyruvic acid | 100±21 | 103±6 | 101±5 | 100±6 | 96±11 | NS |
| Quinolinic acid | 62±5 | 100±12 | 111±20 | 122±19 | 106±30 | NS |
| Sarcosine | 16±5 | 159±80 | 87±35 | 141±79 | 97±57 | NS |
| Sorbitol | 111±11 | 94±6 | 98±5 | 96±2 | 101±6 | NS |
| Spermidine | 126±33 | 80±12 | 110±27 | 84±9 | 100±19 | NS |

Table S6 continued

|  | Casein | Beef  Leg | Pork  Leg | Chicken  Leg | Chicken  Breast | ANOVA |
| --- | --- | --- | --- | --- | --- | --- |
| Stearic acid | 107±8 | 95±3 | 105±4 | 95±3 | 98±5 | NS |
| Sucrose | 96±16 | 93±15 | 94±6 | 106±17 | 111±20 | NS |
| Tartaric acid | 292±235 | 93±69 | 37±9 | 43±13 | 34±9 | NS |
| Thymidine | 101±18 | 83±22 | 88±10 | 128±23 | 100±14 | NS |
| Thymine | 117±17 | 87±5 | 110±6 | 93±8 | 93±8 | NS |
| Tryptamine | 126±32 | 83±12 | 108±27 | 84±10 | 100±17 | NS |
| Tryptophan | 91±23 | 101±22 | 79±31 | 114±8 | 115±25 | NS |
| Tyrosine | 114±15 | 85±14 | 81±16 | 128±9 | 92±15 | NS |
| Uracil | 101±8 | 83±6 | 111±8 | 103±12 | 102±13 | NS |
| Uric acid | 70±13 | 61±16 | 125±19 | 129±22 | 115±41 | NS |
| Uridine | 160±41 | 85±26 | 61±12 | 106±36 | 87±35 | NS |
| Urocanic acid | 98±18 | 103±16 | 104±13 | 116±4 | 78±11 | NS |
| Xanthine | 94±10 | 70±9 | 120±14 | 121±16 | 93±21 | NS |
| Xanthosine | 98±10 | 105±15 | 96±15 | 78±13 | 122±22 | NS |

Relative values are means with their standard errors (n = 6). NS: not significant (P ≥ 0.05); ANOVA: analysis of variance. Different letters in the same line denote significantly different mean values according to the Tukey test (P < 0.05).
