# Supplementary material for: Effects of Dietary Defatted Meat Species on Metabolomic Profiles of Murine Liver, Gastrocnemius Muscle, and Cecal Content
Source: Metabolites. 2020 Dec 9;10(12):503. doi: 10.3390/metabo10120503 (PMC7763243; doi:10.3390/metabo10120503)
Supplement: Supplementary file 1 [file metabolites-10-00503-s001.zip › Supplementary Tables/Table S8 Diet composition.docx]

Table S7 Composition of the experimental diets

| Ingredient (%) | Casein | Beef  Leg | Pork  Leg | Chicken  Leg | Chicken  Breast |
| --- | --- | --- | --- | --- | --- |
| Casein | 20.00 | - | - | - | - |
| Beef leg | - | 20.54 | - | - | - |
| Pork leg | - | - | 20.14 | - | - |
| Chicken leg | - | - | - | 20.24 | - |
| Chicken breast | - | - | - | - | 19.91 |
| Corn starch | 39.75 | 39.31 | 39.81 | 39.71 | 40.04 |
| Soy oil | 7.00 | 6.90 | 6.80 | 6.80 | 6.80 |
| α-Corn starch | 13.20 | 13.20 | 13.20 | 13.20 | 13.20 |
| Cellulose | 5.00 | 5.00 | 5.00 | 5.00 | 5.00 |
| Sucrose | 10.00 | 10.00 | 10.00 | 10.00 | 10.00 |
| Mineral mix  （AIN-93G） | 3.50 | 3.50 | 3.50 | 3.50 | 3.50 |
| Vitamin mix  （AIN-93VX） | 1.00 | 1.00 | 1.00 | 1.00 | 1.00 |
| L-Cystine | 0.30 | 0.30 | 0.30 | 0.30 | 0.30 |
| Choline bitartrate | 0.25 | 0.25 | 0.25 | 0.25 | 0.25 |
| tert-Butylhydroquinone | 0.00 | 0.00 | 0.00 | 0.00 | 0.00 |
