# Supplementary material for: Effects of Dietary Defatted Meat Species on Metabolomic Profiles of Murine Liver, Gastrocnemius Muscle, and Cecal Content
Source: Metabolites. 2020 Dec 9;10(12):503. doi: 10.3390/metabo10120503 (PMC7763243; doi:10.3390/metabo10120503)
Supplement: Supplementary file 1 [file metabolites-10-00503-s001.zip › Supplementary Tables/Table S9 Amino acid composition.docx]

Table S8 Amino acid composition of experimental protein sources

|  |  | Casein | Beef  Leg | Pork  Leg | Chicken  Leg | Chicken  Breast | Requirement |
| --- | --- | --- | --- | --- | --- | --- | --- |
| Essential amino acids | Threonine | 7.8 | 9.3 | 8.6 | 8.3 | 8.5 | 4.0 |
|  | Tryptophan | 1.5 | 1.7 | 1.8 | 1.6 | 1.9 | 1.0 |
|  | Phenylalanine | 9.7 | 8.8 | 8.2 | 7.6 | 7.9 | 7.6 |
|  | Valine | 12.0 | 10.5 | 10.0 | 9.2 | 9.9 | 5.0 |
|  | Leucine | 17.9 | 17.5 | 16.5 | 15.5 | 16.2 | 7.0 |
|  | Isoleucine | 10.0 | 10.0 | 9.4 | 8.7 | 9.6 | 4.0 |
|  | sulfur amino acid | 5.3 | 5.6 | 5.5 | 5.5 | 5.5 | 5.0 |
|  | Lysine | 15.0 | 19.3 | 18.3 | 18.4 | 18.7 | 4.0 |
|  | Histidine | 5.4 | 8.7 | 12.0 | 6.1 | 7.1 | 2.0 |
|  | Arginine | 7.3 | 14.1 | 13.6 | 14.6 | 13.8 | 3.0 |
| Non-essential amino acids | Glycine | 3.2 | 8.2 | 8.4 | 11.3 | 7.8 |  |
|  | Alanine | 5.6 | 11.2 | 10.6 | 11.5 | 10.7 |  |
|  | Serine | 9.9 | 7.4 | 7.3 | 7.5 | 7.1 |  |
|  | Aspartate | 13.7 | 19.8 | 18.9 | 18.5 | 18.8 |  |
|  | Glutamate | 43.4 | 34.5 | 32.2 | 34.6 | 31.7 |  |
|  | Tyrosine | 11.7 | 8.1 | 7.6 | 6.8 | 7.4 |  |
|  | Proline | 20.5 | 7.7 | 7.6 | 9.1 | 6.8 |  |

The unit is g/kg diet
